# Border-Associated Macrophage Migrasomes in Alzheimer’s Disease: An Emerging Aβ–Senescence–Microglia Axis?

**DOI:** 10.3390/cells15181671

**Published:** 2026-09-16

**Authors:** James Chmiel, Marta Kopańska

**Affiliations:** 1 Institute of Physical Culture Sciences, Faculty of Physical Culture and Health, University of Szczecin, Al. Piastów 40B Block 6, 71-065 Szczecin, Poland; 2 Department of Medical Communication and Professional Competency Development, Faculty of Medicine, Collegium Medicum, University of Rzeszów, 35-310 Rzeszów, Poland; mkopanska@ur.edu.pl

**Keywords:** Alzheimer’s disease, border-associated macrophages, migrasomes, amyloid-β, cerebral amyloid angiopathy, microglial senescence, CD5L/AIM, complement activation, blood–brain barrier, neuroinflammation

## Abstract

Alzheimer’s disease (AD) involves not only the neural parenchyma but also brain-border interfaces in which border-associated macrophages (BAMs) regulate amyloid-β (Aβ) handling, vascular function, and immune surveillance. This review focuses on a pathological axis in which persistent vascular Aβ40 and aging-related stress shift clearance-competent BAMs toward oxidative-stress and senescent-like states. Two experimentally supported but incompletely connected branches are emphasized. In one, Aβ engages macrophage CD36–NOX2 signaling and generates reactive oxygen species that impair neurovascular function. In the other, Aβ40 internalization promotes TSPAN4-dependent migrasome formation and enrichment of CD5L/AIM; vascularly deposited CD5L/AIM lowers endothelial CD59, facilitates C5b–9 formation, and damages the blood–brain barrier. A separate aging study indicates that CD5L/AIM-rich BAM migrasomes can transmit apoptosis resistance and senescence-like dysfunction to microglia. Human evidence is currently strongest for CAA rather than parenchymal AD: a small CAA cohort showed increased circulating CD14-positive migrasomes and monocyte TSPAN4, with an exploratory area under the ROC curve of approximately 0.91 for TSPAN4-positive monocytes versus healthy controls, whereas AD patients selected to lack imaging evidence of CAA did not show increased circulating migrasome counts. Accordingly, the Aβ40–TSPAN4–CD5L/AIM vascular branch should presently be regarded as a CAA-enriched mechanism that may be especially relevant to AD with prominent CAA, not as a universal mechanism of Aβ42-dominant sporadic AD without substantial vascular amyloid. Direct demonstration of BAM-derived migrasomes in human AD brain tissue is still lacking, and neither TSPAN4 nor CD5L/AIM is sufficiently specific to serve as a stand-alone biomarker. We therefore distinguish peer-reviewed human observations, experimental causal evidence, preprint findings, and proposed cross-pathway interactions and outline biomarker validation and pathway-selective therapeutic strategies that preserve beneficial BAM functions.

## 1. Why Brain Borders Matter in Alzheimer’s Disease

Alzheimer’s disease (AD) is classically defined by amyloid-β (Aβ) deposition, tau pathology, synaptic dysfunction, and neurodegeneration, but these lesions develop within a larger brain-border system that regulates exchange among neural tissue, blood, cerebrospinal fluid (CSF), meninges, lymphatic vessels, choroid plexus, and cranial bone marrow. The blood–brain barrier (BBB), perivascular and leptomeningeal spaces, meningeal lymphatics, choroid plexus, and skull–dura channels are therefore active components of molecular clearance and immune surveillance rather than passive anatomical boundaries [1,2,3,4,5,6].

This perspective is directly relevant to Aβ because extracellular peptide burden depends on degradation, cellular uptake, vascular transport, perivascular movement, exchange with CSF, and downstream lymphatic drainage. Experimental work shows that periarterial/paravascular transport and aquaporin-4 polarization decline with aging, while parenchymal border macrophages can modify perivascular extracellular matrix and CSF-flow resistance. These observations place border macrophages at a site where impaired clearance can increase local Aβ exposure and where macrophage dysfunction can, in turn, worsen clearance [7,8,9,10,11].

The BBB provides a second mechanistic link. Human imaging and biomarker studies demonstrate age- and cognitive-impairment-associated barrier leakage, and APOE4 can promote neurovascular injury through pericyte and BAM-associated pathways. In experimental humanized APOE models, BAM-derived APOE4 increases calcium- and NADPH-oxidase-dependent vascular oxidative stress, impaired neurovascular coupling, and reduced cerebral perfusion. These data support the concept that genetically and amyloid-sensitized BAMs can participate in vascular dysfunction before end-stage tissue injury [12,13,14,15,16,17].

Other border compartments influence the same clearance environment. Meningeal lymphatic impairment aggravates Aβ accumulation and inflammatory responses in AD models, whereas lymphatic enhancement can improve drainage and the efficacy of anti-Aβ antibodies. The choroid plexus contributes to Aβ handling at the blood–CSF interface, and skull–dura vascular channels connect cranial marrow with meningeal immune compartments. Together, these routes create a spatially connected system in which vascular amyloid, inflammatory mediators, CSF signals, and locally supplied myeloid cells can influence one another [18,19,20,21,22,23,24,25].

BAMs occupy key positions within this system. Experimental studies indicate that they can support perivascular Aβ clearance, yet chronic amyloid exposure can also recruit macrophage CD36–NOX2 signaling, reactive oxygen species (ROS), and CAA-promoting vascular dysfunction. Thus, the relevant disease transition is not a simple switch from a universally beneficial to a universally harmful macrophage population, but a change in subset, anatomical niche, and functional state under sustained amyloid and vascular stress [26,27,28].

This distinction is important during anti-Aβ immunotherapy. Mobilized Aβ must be processed through plaque-associated, vascular, and border compartments; in mouse models, antibody-associated microhemorrhage is accompanied by activation of perivascular macrophages and recruitment of peripheral monocytes. These findings do not make BAM activation synonymous with toxicity, but they show that the vascular immune environment can influence whether amyloid mobilization is resolved safely or accompanied by permeability changes, complement activation, and hemorrhagic injury [29].

Human evidence supports the existence of specialized border myeloid states but remains less mechanistically resolved than the animal literature. Single-cell profiling of human leptomeninges has identified transcriptionally distinct BAM populations and AD-associated changes in macrophage–fibroblast signaling, while spatial proteomic analysis of human AD cortex has identified perivascular macrophages and BAM-like myeloid cells enriched in plaque- and vessel-related niches. Because activated microglia and BAMs share several markers, however, postmortem protein expression alone cannot establish developmental identity or prove that a specific human BAM subset produced a migrasome [6,30].

**Figure 1:**
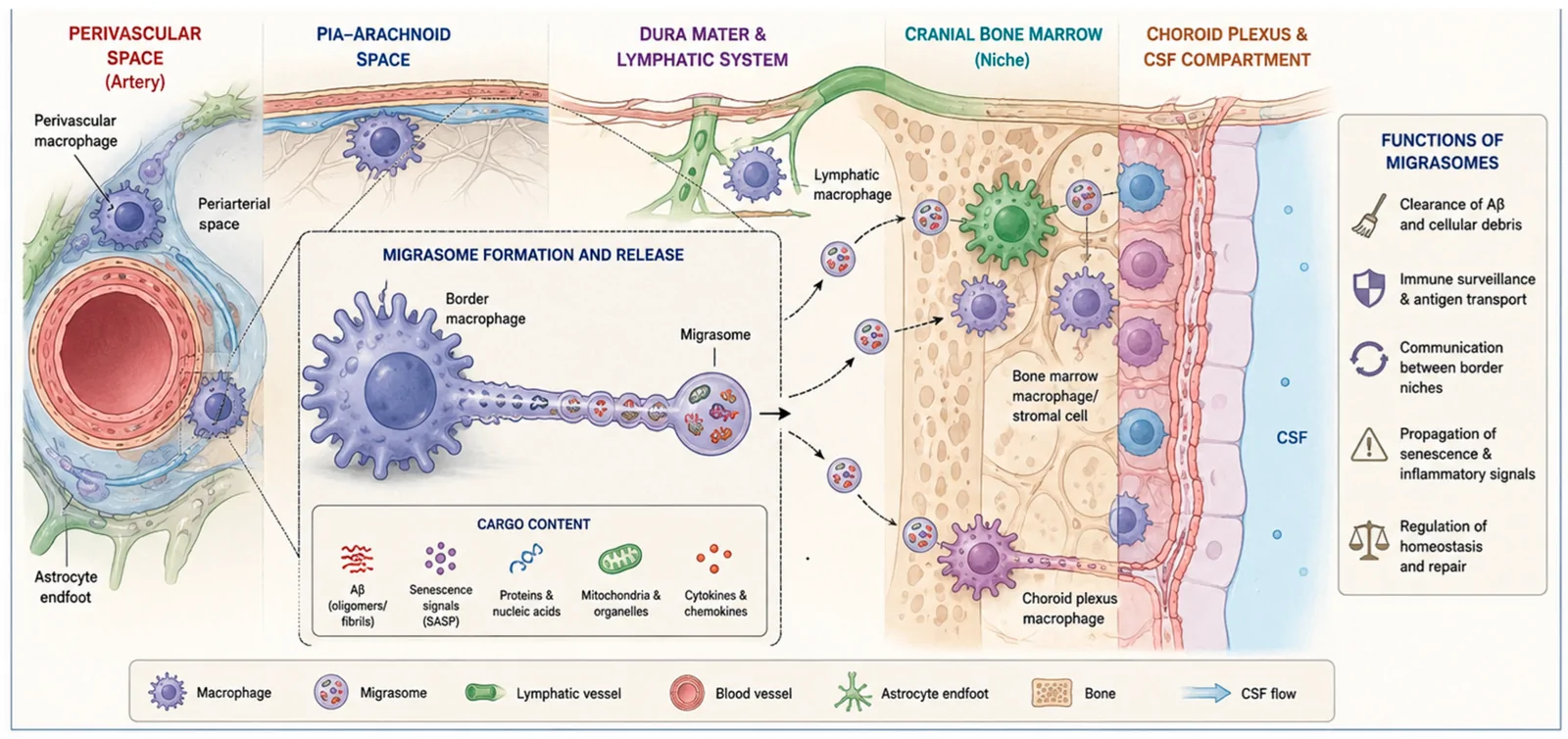
Pathology-focused model of border-associated macrophage (BAM)-derived migrasomes within the brain-border network. BAMs are distributed across perivascular, leptomeningeal, dural, and choroid-plexus compartments. Persistent vascular Aβ40, oxidative stress, aging-related metabolic dysfunction, and complement exposure may shift selected BAM populations from clearance-competent states toward oxidative-stress or senescent-like states. TSPAN4-dependent migrasomes can then concentrate CD5L/AIM and other cargo at vascular surfaces or deliver senescence-associated signals to nearby cells. The experimentally supported vascular branch links Aβ40-induced migrasomes with reduced endothelial CD59, C5b–9 formation, and BBB injury; the aging branch links BAM migrasomes with CD5L/AIM-dependent microglial apoptosis resistance and senescence-like dysfunction. Direct trafficking among all illustrated border compartments, and direct demonstration of BAM-derived migrasomes in human AD brain tissue, remain unverified.

The central model examined in this review is therefore deliberately narrow: persistent Aβ40 and aging-related stress may drive clearance-competent BAMs toward an oxidative-stress state and/or a senescent-like, migrasome-producing state, with downstream effects on the BBB, complement system, and microglia. Throughout the review, ‘demonstrated’ or ‘established’ is reserved for directly tested relationships, ‘human-associated’ for observational findings in human samples, and ‘proposed’ or ‘hypothetical’ for mechanistic links not yet tested directly. Most causal steps remain derived from cell and mouse experiments, and direct evidence for BAM-derived migrasomes in human AD brain tissue is not yet available. Pathology-focused model of BAM-derived migrasomes within the brain-border network is presented in Figure 1.

### Literature Search and Review Approach

This narrative review was developed through a structured literature search focused on border-associated macrophages, migrasome biology, cerebral amyloid angiopathy, Alzheimer’s disease, cellular senescence, complement activation, and neurovascular dysfunction. PubMed/MEDLINE, Web of Science, and Scopus were searched for English-language publications available up to [DATE]. Search terms included combinations of “border-associated macrophages”, “perivascular macrophages”, “CNS-associated macrophages”, “migrasomes”, “migracytosis”, “TSPAN4”, “CD5L”, “AIM”, “amyloid beta”, “Aβ40”, “Aβ42”, “cerebral amyloid angiopathy”, “Alzheimer’s disease”, “microglia”, “senescence”, “complement”, and “blood–brain barrier”. Reference lists of relevant primary studies and reviews were additionally screened. Priority was given to peer-reviewed primary mechanistic studies, human observational studies, and recent high-quality reviews. Preprints were included only when directly relevant to emerging BAM biology, were explicitly identified as non-peer-reviewed evidence, and were not used as the sole basis for definitive mechanistic conclusions. Because the purpose of the article was conceptual integration rather than exhaustive quantitative evidence synthesis, study selection and interpretation followed a narrative-review framework, with attention to SANRA principles.

## 2. Identity and Heterogeneity of Border-Associated Macrophages

Border-associated macrophages (BAMs), also termed central nervous system-associated macrophages, comprise resident macrophage populations positioned outside the neural parenchyma at the interfaces between the brain, CSF, meninges, vasculature, choroid plexus, and skull. The term encompasses macrophages of the dura mater, leptomeninges, perivascular spaces, and choroid plexus. However, BAMs should not be treated as a single cell type. Their developmental origin, turnover, transcriptomic identity, access to circulating molecules, exposure to CSF, and interactions with endothelial, stromal, epithelial, and vascular cells differ substantially between anatomical compartments. Single-cell and spatial studies therefore support a hierarchical model in which BAMs share a core macrophage identity but differentiate into anatomically and functionally specialized subsets [4,31,32,33,34].

BAMs must first be distinguished from parenchymal microglia. Both populations express general myeloid and macrophage genes, including AIF1, CSF1R, CX3CR1, C1QA, C1QB, C1QC, FCER1G, and TYROBP, and both can display phagocytic, lipid-processing, complement, inflammatory, and antigen-presentation programs. Nevertheless, homeostatic microglia are preferentially characterized by genes such as P2RY12, TMEM119, HEXB, SALL1, SLC2A5, and GPR34, whereas BAMs are commonly enriched for MRC1, encoding CD206, LYVE1, F13A1, STAB1, CD163, and MS4A7. Human multiomic profiling identified MRC1, F13A1, STAB1, and CD163 as relatively conserved BAM-associated genes across species. In contrast, P2RY12 and related homeostatic markers remained more closely associated with microglia [4,35].

These marker combinations are useful but not absolute. CD206 and CD163 are expressed by many BAMs but may differ quantitatively between compartments and states. LYVE1 identifies major perivascular and leptomeningeal populations in mice but is neither uniformly expressed by every BAM nor entirely restricted to BAMs outside the CNS. CX3CR1 is shared with microglia and can be lost by particular perivascular macrophage subsets during maturation. Conversely, microglial markers such as P2RY12 and TMEM119 can be reduced during neurodegeneration, while activated BAMs may acquire APOE-, SPP1-, complement-, or lipid-associated programs resembling disease-associated microglia. Cell identity should therefore be assigned using the combination of anatomical localization, multiple molecular markers, transcriptional profile, and, where possible, developmental lineage rather than any single protein [4,35,36].

The distinction from circulating monocytes and monocyte-derived macrophages is equally important. BAMs were once assumed to be continuously replaced by blood monocytes because of their proximity to fenestrated vessels and peripheral immune compartments. Fate-mapping experiments subsequently demonstrated that substantial BAM populations are established from embryonic precursors and can persist through local self-renewal. However, the extent of peripheral replacement varies among anatomical niches and can increase with age, inflammation, depletion, irradiation, or neurological disease. Monocyte-derived cells entering a border compartment can eventually acquire BAM-like morphology and parts of the resident transcriptional program, but their developmental history may leave persistent molecular and functional differences. The BAM compartment is therefore composed not only of spatially distinct subsets but, under some conditions, of cells with different ontogenetic origins occupying the same niche [4,31,32,37].

Early fate-mapping studies established that microglia and many non-parenchymal CNS macrophages originate from primitive yolk-sac hematopoiesis rather than adult bone marrow [31]. Subsequent work refined this model by showing that microglial and BAM development diverges surprisingly early. Utz and colleagues identified phenotypically and transcriptionally distinct macrophage populations in the developing brain that independently generated microglia and BAMs. Microglial specification depended strongly on transforming growth factor-β-related developmental signaling, whereas the early BAM lineage was comparatively independent of this microglial differentiation program [32]. The distinction is functionally important because suppression of microglial specification can cause embryonic brain macrophages to acquire BAM-associated features rather than simply producing incompletely developed microglia [32,37].

Developmental origin alone, however, does not fully determine mature identity. Masuda and colleagues demonstrated that microglia and meningeal macrophages share a prenatal progenitor, whereas perivascular macrophages arise predominantly after birth from perinatal meningeal macrophages. Their migration into the developing perivascular space required integrin signaling and the presence of arterial vascular smooth-muscle cells. Thus, perivascular macrophage identity is generated through an interaction between lineage history and a specific postnatal vascular niche. BAM heterogeneity is not simply inherited from multiple progenitors; it is progressively imposed by anatomical microenvironments [34].

The dura mater contains one of the most peripherally accessible BAM compartments. Dural macrophages reside outside the arachnoid barrier, near fenestrated dural vessels, venous sinuses, lymphatic vessels, and channels communicating with skull bone marrow. Compared with macrophages at more deeply protected CNS interfaces, dural BAMs are exposed more directly to blood-borne molecules and locally supplied hematopoietic cells. They commonly display relatively strong antigen-processing and major histocompatibility complex class II programs, although the population also contains scavenging and tissue-remodeling states. Dural preparations can additionally contain conventional dendritic cells and recently recruited monocytes, making anatomical and molecular separation essential. Human interface profiling identified a dura-associated monocyte-derived macrophage population characterized by genes involved in cellular recruitment and tissue homing, further demonstrating that not every macrophage-like cell in the dura is a long-lived resident BAM [23,35].

Leptomeningeal macrophages occupy the pia–arachnoid compartment and the CSF-filled subarachnoid space. They are positioned beside leptomeningeal vessels, fibroblasts, arachnoid trabeculae, nerves, and CSF-borne molecular signals. These cells share several markers with perivascular macrophages, including CD206, CD163, LYVE1, F13A1, and scavenger-receptor programs, but they remain transcriptionally distinguishable from microglia. Their position permits simultaneous sampling of CSF-associated antigens and interaction with vascular and stromal cells. The leptomeningeal compartment may also act as a developmental source for perivascular macrophages, supporting a close ontogenetic relationship between these populations despite their different mature locations [6,34,35].

Perivascular macrophages reside within the spaces surrounding penetrating cerebral vessels, anatomically positioned between vascular and glial basement-membrane compartments. They should be distinguished from pericytes, vascular smooth-muscle cells, endothelial cells, perivascular fibroblasts, and parenchymal microglia that extend processes toward the vessel wall. In mice, major perivascular populations express LYVE1, F4/80, CD206, CD64, CD163, and variable CX3CR1. Most parenchymal perivascular macrophages examined by Siret and colleagues were LYVE1-positive, MHC class II-negative, and CD45-low or intermediate. The same study identified both conventional CX3CR1-positive and previously underrecognized CX3CR1-negative perivascular macrophages. The latter derived from CX3CR1-expressing precursors, retained phagocytic activity, and accounted for a substantial minority of the LYVE1-positive population in adult mice before declining with age [36].

Human perivascular BAMs likewise have a distinctive phenotype. Multiomic analysis demonstrated co-expression of CD206 and CD163 across several human BAM compartments, while CD169 was most strongly represented in perivascular BAMs. This expression pattern is consistent with an enhanced scavenging phenotype at the vascular interface. Perivascular cells also displayed weaker MHC class II-associated programs than BAMs from the choroid plexus, leptomeninges, and dura, although these differences represent relative enrichment rather than rigid categories. The phenotype of a perivascular BAM is consequently shaped both by its core macrophage program and by its association with particular vessel types, basement membranes, extracellular-matrix components, and transported molecules [35].

Choroid-plexus macrophages constitute an especially diverse group because the choroid plexus contains several physically separated macrophage niches. Stromal macrophages are located within the vascularized connective-tissue core and are exposed to fenestrated blood vessels. Other macrophage-like cells are positioned near the epithelial layer or on its ventricular surface. Epiplexus macrophages, historically termed Kolmer cells, face the CSF and display a phenotype that is more microglia-like than that of conventional stromal BAMs. Single-cell and fate-mapping studies have identified an apically located choroid-plexus population with microglial characteristics, while human profiling showed that Kolmer cells expressed BHLHE41, APOE, SPP1, and TTR and clustered closer to activated microglial states than to CD206-positive stromal BAMs [4,5,35].

Choroid-plexus macrophages also exhibit greater ontogenetic and turnover complexity than many leptomeningeal and perivascular macrophages. The choroid plexus contains long-lived embryonically derived cells, microglia-like epiplexus cells, and macrophages that can be replenished from circulating precursors. Its macrophage composition changes during development, aging, and inflammation, while the cellular environment differs between the lateral, third, and fourth ventricles. Consequently, studies that pool an entire choroid plexus may combine macrophages with different access to blood, CSF, epithelium, extracellular matrix, and ventricular signals. Such pooling can obscure disease effects that are restricted to stromal, epithelial-associated, or epiplexus populations [4,5,35].

A second classification system divides selected murine BAM populations into CD206-low/MHC class II-high and CD206-high/MHC class II-low states, sometimes termed BAM1 and BAM2. These labels are study-specific and should not be treated as a universal binary taxonomy. Human studies have also used BAM_1, BAM_2, and BAM_3 for transcriptionally defined clusters that are not equivalent to the murine CD206/MHC class II scheme. To avoid conflation, this review uses anatomical labels whenever possible and uses functional descriptors only when supported by the cited experiment: ‘clearance-competent/homeostatic-like BAMs’ for states retaining scavenging and vascular-support functions, ‘oxidative-stress BAMs’ for the Aβ–CD36–NOX2 phenotype, and ‘senescent-like BAMs’ for cells displaying the senescence-associated features reported in the aging study. BAM1/BAM2 terminology is retained only when discussing studies that explicitly used those labels [4,6].

Disease further expands BAM heterogeneity. Resident BAMs can alter antigen presentation, complement activity, lipid metabolism, oxidative-stress responses, phagocytosis, cytokine signaling, and extracellular-matrix interactions. Simultaneously, CCR2-positive monocytes can enter border niches and become macrophages with partial BAM-like characteristics. In experimental neuroinflammation, recruited monocyte-derived macrophages displayed greater transcriptional plasticity than resident BAMs and adopted inflammatory programs that were not completely reproduced by the embryonically derived population. More recent lineage-tracing experiments showed that CCR2-positive monocytes can persist and replenish BAM-like populations in diseased mouse brains, demonstrating that pathological border compartments may contain a mixture of resident and recruited macrophages rather than a uniformly transformed resident population [38,39].

This distinction has substantial implications for AD research. A macrophage identified near an amyloid-laden vessel or plaque may represent an embryonically derived perivascular BAM, a leptomeningeal macrophage extending into a vascular compartment, a recently recruited monocyte-derived macrophage, or a parenchymal microglial cell adjacent to the vascular basement membrane. These cells can express overlapping markers after exposure to Aβ, APOE, lipids, complement, or inflammatory cytokines. Studies based only on IBA1, CD68, CX3CR1, or morphology cannot resolve these alternatives reliably. Even CD206 and CD163 should be combined with vascular localization, microglial-exclusion markers, lineage-sensitive tools, or spatial transcriptomic information.

Human data confirm that BAM heterogeneity is relevant to AD rather than merely a feature of healthy mouse anatomy. Single-nucleus profiling of aged human leptomeninges identified three transcriptionally distinct BAM populations. All expressed CD163, whereas one subtype was distinguished by CD83 expression. Collectively, leptomeningeal BAMs differed from parenchymal microglia by approximately 780 genes, indicating that human BAM identity cannot be reduced to an activated microglial profile. AD-risk genes were enriched in both microglia and BAMs but were distributed differently: MS4A4A, CD33, and CR1 were prominent in one BAM population; HLA genes and PTK2B were enriched in another; and APOE, TOMM40, and TREM2 were shared between microglia and one BAM subtype [6].

The same study found particularly extensive AD-associated transcriptional changes in leptomeningeal BAMs and fibroblasts. Altered signaling involved extracellular-matrix, complement, APP, SPP1, MHC class I, and TGF-β-related pathways, suggesting that BAM dysfunction in AD may emerge through interaction with the surrounding stromal niche rather than through an exclusively cell-autonomous immune process. These observations are important because the senescence and migrasome phenotypes proposed for BAMs may depend on local collagen, laminin, vascular, and fibroblast-derived signals. A BAM exposed to vascular Aβ in a perivascular basement membrane may consequently respond differently from a dural BAM exposed to skull-marrow-derived immune signals or a choroid-plexus macrophage exposed to CSF and fenestrated blood vessels [6].

A 2026 bioRxiv preprint provides a further AD-specific example of functional BAM heterogeneity. The study separated CD206-low/MHC class II-high BAM1 cells from CD206-high/MHC class II-low BAM2 cells and reported that BAM2 cells possessed strong phagocytic and metabolic characteristics. BAM2 depletion accelerated early abnormalities in 5×FAD mice, whereas BAM2 abundance and metabolic capacity declined as amyloid pathology progressed; analyses of human AD datasets and tissue were reported to show concordant BAM loss and metabolic impairment. Because these findings remain a preprint and have not undergone journal peer review, they are treated here as provisional evidence and are not used as sole support for a verified human mechanism [40].

The identity of the senescent, migrasome-producing macrophages described during aging and amyloid-related vascular injury must therefore be defined more precisely. It remains uncertain whether migrasome production is concentrated in CD206-high scavenging perivascular macrophages, MHC class II-high antigen-presenting BAMs, monocyte-derived macrophages, or a senescent state shared across several border populations. Similarly, CD5L/AIM expression, TSPAN4-dependent migrasome formation, complement activation, and communication with microglia may vary by anatomical niche and disease stage. Referring broadly to “BAM-derived migrasomes” is useful as an initial framework, but mechanistic studies should establish the exact lineage, location, phenotype, and turnover of the producing cells.

Recent depletion experiments demonstrate that meningeal macrophage populations are not functionally interchangeable. Lohat and colleagues found that broad macrophage depletion affecting leptomeningeal macrophages disrupted cerebrospinal-fluid drainage to meningeal lymphatic vessels and was accompanied by reduced lymphatic-vessel density. Although dural macrophages and the meningeal lymphatic network recovered during the subsequent two weeks, leptomeningeal macrophage abundance and efficient CSF drainage remained impaired. In contrast, selective depletion of dural macrophages using a systemically administered CSF1R-targeting antibody did not substantially disrupt meningeal lymphatic structure or CSF drainage. These results identify leptomeningeal macrophages as particularly important cellular regulators of CSF transfer to meningeal lymphatic vessels. They also reinforce the need to establish whether pathological migrasomes are produced by leptomeningeal, perivascular, dural, or several BAM populations, because identical migrasomal cargo could have different consequences depending on the anatomical site of deposition [41].

BAM heterogeneity should therefore be interpreted along anatomical, ontogenetic, functional, disease-state, and temporal dimensions. The central question is not whether BAMs are globally beneficial or harmful, but which subset performs which function at a given border and disease stage. In the remainder of this review, evidence from peer-reviewed human samples is separated from animal/cell perturbation studies and from the 2026 preprint [40]; hypothetical cross-links are explicitly identified as such. This framework is essential for interpreting the proposed BAM–migrasome–microglia axis and for designing interventions that suppress pathological signaling without eliminating beneficial clearance and vascular-support functions.

## 3. Migrasomes as a Pathology-Relevant BAM Signaling Mechanism

Migrasomes are large membrane-bound structures that form on retraction fibers generated by migrating cells. Their defining features include migration/retraction-fiber dependence, characteristic morphology with intraluminal vesicles, and enrichment of tetraspanins and other migrasome-associated proteins. TSPAN4 is a strong promoter of migrasome biogenesis but is not a unique migrasome marker; convincing identification, especially in fixed human tissue or plasma, should combine morphology with multiple molecular markers such as TSPAN4 and migrasome-enriched proteins including NDST1, PIGK, CPQ, or EOGT, together with evidence of cellular origin whenever possible [42,43,44,45,46,47].

### 3.1. Biogenesis Is Coupled to Migration, Extracellular Matrix, and Membrane Organization

Migrasome formation is spatially constrained by retraction-fiber adhesion to extracellular matrix. Integrin–ligand pairing helps determine where migrasomes form, while migration speed and persistence influence retraction-fiber architecture and migrasome output. Rab35-dependent integrin organization, TSPAN4/cholesterol-rich membrane domains, sphingomyelin synthesis, calcium-sensitive membrane expansion, and GPI-anchored protein nanoclusters contribute to the generation and stabilization of migrasomal structures. Non-canonical migracytosis can also occur under intense cytoskeletal stress, indicating that pathological membrane retraction may sometimes substitute for sustained directional migration. For BAMs, these mechanisms are relevant because vascular basement membranes, perivascular matrix composition, cell motility, and oxidative or inflammatory stress can all alter the physical conditions under which migrasomes are deposited [43,44,48,49,50,51,52,53].

### 3.2. Cargo Loading Enables Spatially Concentrated Pathological Signaling

Migrasomes can carry proteins, messenger RNAs, smaller intraluminal vesicles, and conventionally secreted signaling molecules. Experimental studies have shown transfer of functional RNA/protein cargo, Rab10–caveolin-1-dependent delivery of intraluminal vesicles, and redirection of secretory carriers into migrasomes. The feature most relevant to AD is spatial concentration: an intact migrasome can remain at or dock to a discrete extracellular site, protecting and concentrating cargo in a local microdomain rather than allowing immediate dilution in blood or CSF. In the CAA model discussed below, this property is critical because soluble CD5L/AIM at circulating concentrations was insufficient to reproduce endothelial injury, whereas migrasome-mediated vascular enrichment increased local exposure [54,55,56,57].

### 3.3. In Vivo Evidence Is Retained Only Insofar as It Informs the BAM–AD Axis

Studies in development, angiogenesis, germ-cell migration, mitochondrial disposal, and coagulation establish that migrasomes can function as stable in vivo cargo-deposition platforms, but those physiological roles are not central to the present review and are not considered further [58,59,60,61,62]. In the nervous system, work in experimental stroke supports the broader principle that migrasome-related signaling can alter injury responses [63]. The disease-specific evidence relevant here is more direct: in CAA, Aβ40-stimulated macrophage-lineage cells generate TSPAN4-positive, CD5L/AIM-enriched migrasomes that attach to vascular surfaces and facilitate complement-dependent BBB injury [57]; in aging mice, senescent-like BAMs release CD5L/AIM-containing migrasomes that are taken up by microglia and promote apoptosis resistance and secondary senescence-like dysfunction [64].

### 3.4. Human Evidence and Translational Boundary

The 2023 CAA study provides the only direct human-associated migrasome evidence that anchors the proposed AD/CAA mechanism. It identified migrasome-like structures around skin vessels from a CAA patient, increased plasma migrasome abundance and CD14-positive migrasomes in a small CAA cohort, and increased monocyte expression of TSPAN4 and other migrasome-associated markers [57]. These observations are clinically relevant but do not constitute demonstration of BAM-derived migrasomes in human brain tissue. The mechanistic steps linking Aβ40 uptake, TSPAN4-dependent migrasome production, CD5L/AIM loading, CD59 reduction, complement activation, and BBB injury were established primarily in cultured cells and mouse models. Likewise, the BAM-to-microglia senescence pathway was demonstrated in aged mice rather than in biomarker-defined human AD [64]. The two central mechanistic reports also originated from the same research group with overlapping authors, so independent replication is required before the complete pathway can be considered established. Table 1 presents experimental and translational evidence supporting the proposed BAM–migrasome axis.

Collectively, the complete proposed axis has not been demonstrated within a single experimental model or in human AD; rather, it represents an integration of partially connected mechanistic observations of unequal evidential strength.

## 4. Amyloid-β as a Driver of BAM Dysfunction

Amyloid-β (Aβ) is usually discussed as a neuronal and parenchymal proteinopathy, but its pathological effects extend far beyond plaques. Soluble Aβ released into the interstitial fluid must cross or travel along several brain-border compartments before it can be eliminated. These routes include transport across the blood–brain barrier, movement through perivascular basement membranes, exchange with cerebrospinal fluid, uptake by vascular and immune cells, and downstream lymphatic drainage. BAMs, particularly perivascular and leptomeningeal macrophages, are positioned directly within these pathways. They are therefore exposed not only to deposited vascular amyloid but also to soluble, oligomeric, antibody-bound, and cell-associated Aβ moving out of the parenchyma. This anatomical arrangement makes BAMs important participants in Aβ clearance while simultaneously exposing them to chronic proteotoxic, oxidative, metabolic, and inflammatory stress [11,26,57,64,65,66].

The available evidence supports a stage-dependent model rather than a simple harmful-versus-protective classification. Initially, BAMs can internalize and remove Aβ from perivascular and vascular compartments. As exposure persists, however, uptake may exceed degradative capacity, alter receptor signaling, impair mitochondrial and lysosomal function, increase reactive oxygen species production, and change the structures and mediators released by BAMs. Chronically exposed BAMs may consequently shift from Aβ-clearing cells toward metabolically exhausted, inflammatory, senescent-like, or migrasome-producing states. Aβ should therefore be considered both a cargo handled by BAMs and a signal that reprograms BAM identity and function.

### 4.1. BAMs Encounter Distinct Pools of Amyloid-β at Brain Borders

Aβ peptides differ in their biochemical behavior and anatomical distribution. Aβ42 is relatively aggregation-prone and is strongly represented in parenchymal plaques, whereas Aβ40 is more soluble and is the major species associated with leptomeningeal and cortical vascular deposits. CAA and parenchymal AD therefore share Aβ biology but are not interchangeable disease compartments. Their pathogenic pathways intersect through Aβ production, interstitial-fluid transport, and perivascular clearance, yet diverge in the site of deposition and mechanisms of tissue injury [28,57,65,67,68]. This distinction is particularly important for BAM biology. Perivascular and leptomeningeal macrophages are more directly exposed to Aβ40 accumulating along vessel walls and perivascular drainage routes, although they may also encounter soluble or oligomeric Aβ42 arriving from the parenchyma or cerebrospinal fluid. The response of a BAM is therefore likely to depend on peptide species, aggregation state, concentration, duration of exposure, association with APOE or antibodies, and the anatomical compartment in which the encounter occurs.

Experimental tracer and neuropathological studies indicate that soluble material can leave brain tissue along basement membranes associated with capillaries and arteries. Failure or slowing of this pathway favors the retention of Aβ within vessel walls and the development of cerebral amyloid angiopathy (CAA). BAMs located beside these vessels are therefore positioned at a clearance bottleneck. When drainage is effective, they may capture and process Aβ passing through the perivascular compartment. When drainage fails, the same cells are exposed repeatedly to increasing concentrations of vascular amyloid, damaged extracellular matrix, oxidized lipids, complement components, endothelial signals, and blood-derived proteins entering through a compromised barrier [25,65].

This exposure is spatially heterogeneous. Leptomeningeal BAMs encounter amyloid deposited in surface arteries and arterioles, whereas perivascular BAMs associated with penetrating vessels encounter Aβ within deeper vascular and basement-membrane compartments. Choroid-plexus macrophages may instead respond to Aβ in blood or CSF, and dural BAMs may receive amyloid-related signals indirectly through meningeal fluid drainage or local inflammatory communication. Consequently, evidence derived from perivascular macrophages in CAA should not automatically be generalized to all BAM populations or to all sporadic AD. At present, the strongest causal evidence linking Aβ directly to BAM dysfunction concerns perivascular and leptomeningeal macrophages at the neurovascular interface.

The vascular-versus-parenchymal distinction is also evident in human biochemical and epidemiological data. In a classic biochemical analysis, AD brains with minimal congophilic angiopathy contained predominantly Aβ42(43), whereas increasing vascular amyloid was accompanied by a large additional Aβ40 pool concentrated in vessel walls [69]. CAA is common in AD but not obligatory: a systematic review and meta-analysis of 170 studies estimated moderate-to-severe CAA pathology in approximately 48% of AD cases, while MRI-defined lobar microbleeds identified a substantially smaller fraction [70]. These data argue for explicit disease stratification. The Aβ40–TSPAN4–CD5L/AIM migrasome–complement branch is currently expected to operate most strongly in sporadic CAA and in AD with prominent CAA or vascular Aβ40. Its operation in Aβ42-dominant parenchymal AD without substantial CAA remains unproven. Parenchymal AD may still influence BAMs through soluble Aβ reaching drainage routes, age-related metabolic stress, or the BAM–microglia senescence branch, but those possibilities should not be used to infer that the vascular migrasome mechanism is universal across AD.

### 4.2. Early BAM Responses Can Support Amyloid Clearance

The protective capacity of perivascular macrophages was demonstrated by Hawkes and McLaurin in the TgCRND8 model. Experimental depletion of these cells increased the number of amyloid-positive cortical vessels and elevated vascular Aβ, whereas stimulation of perivascular-macrophage turnover reduced CAA. The effect was not explained by corresponding changes in microglial or astrocytic clearance, supporting a specific contribution of the perivascular macrophage compartment. These findings established that BAMs can restrict vascular amyloid accumulation under appropriate functional conditions [26].

Scavenger receptor class B type I, or SR-BI, provides one molecular mechanism supporting this response. In J20 amyloid-model mice, SR-BI was expressed by CD206-positive perivascular macrophages and was closely associated with vascular Aβ deposits. Reduction in SR-BI increased fibrillar plaque burden, aggravated CAA, and worsened learning and memory deficits. Interestingly, reduced SR-BI did not substantially alter Aβ uptake by cultured peripheral macrophages or neonatal microglia, suggesting that its effect may involve the maintenance, positioning, lipid metabolism, or in vivo response of perivascular macrophages rather than simple ligand internalization alone. Increased BAM-marker expression after SR-BI reduction may represent an ineffective compensatory expansion of a functionally impaired population [71].

Recent data further support the existence of a strongly phagocytic BAM subset. In a 2026 preprint, CD206-high, LYVE1-associated BAM2 cells internalized more experimental Aβ per cell than microglia in healthy mice. Selective BAM depletion in the 5×FAD model increased CAA, total amyloid burden, lysosomal abnormalities, neurodegenerative changes, and early memory deficits. Human postmortem and mouse analyses in the same report suggested that BAM numbers decline during AD and that the remaining cells progressively lose endocytic and metabolic competence. Because these findings have not yet completed peer review, they should be treated as compelling but provisional evidence that amyloidogenic conditions exhaust a normally protective BAM population [40].

Together, these studies suggest that BAM abundance alone is not an adequate measure of protective function. Increasing the number of macrophages does not necessarily improve Aβ removal if receptor activity, metabolism, lysosomal degradation, or migration is impaired. A compensatory increase in BAMs may even indicate that the local clearance system is failing. The relevant biological variables include the amount of Aβ internalized per cell, the fate of internalized material, the capacity to export degradation products, and whether the BAM remains viable and functionally stable after repeated exposure.

### 4.3. Aβ Uptake Can Become a Source of Cellular Overload

Phagocytosis protects tissue only when uptake is followed by efficient intracellular processing. Aβ is difficult to degrade because it can oligomerize, form β-sheet-rich fibrils, resist proteolysis, disrupt membranes, and accumulate in endolysosomal compartments. Repeated uptake may therefore transform BAMs into storage sites for incompletely degraded amyloid. As degradative capacity becomes saturated, internalized Aβ may promote lysosomal membrane stress, defective autophagic flux, altered lipid handling, mitochondrial dysfunction, and activation of inflammatory signaling. Direct examination of these processes in molecularly defined BAM subsets remains limited, but several lines of evidence indicate that macrophage handling of Aβ becomes less effective with disease progression [40,57,66].

Zaghi and colleagues compared macrophages obtained from patients with AD and controls. AD-derived macrophages internalized and cleared less Aβ and were more susceptible to apoptosis after exposure to soluble, protofibrillar, or fibrillar forms. Aβ-loaded macrophages adhered to brain endothelial cells and displayed impaired transendothelial migration. In AD brain sections, apoptotic macrophages containing oligomeric Aβ were observed around and within amyloid-laden microvessels. The authors proposed that macrophages may collect Aβ from neural tissue but, after becoming engorged and undergoing apoptosis near vessels, release amyloid that contributes to CAA [66].

These experiments mainly involved blood-derived monocytes and macrophages and should not be interpreted as definitive lineage-specific evidence for resident BAMs. Nevertheless, they illustrate a mechanism highly relevant to BAM dysfunction: a macrophage may begin as a clearance cell but become unable to complete degradation and export. Its retention, death, or rupture at the vessel wall can then return Aβ to the extracellular environment in a more concentrated or aggregated form. The outcome of phagocytosis is thus determined not by internalization alone, but by the entire sequence of uptake, trafficking, degradation, migration, and survival.

The 2026 BAM2 preprint provides more direct evidence for metabolic exhaustion within resident border macrophages. During progression in 5×FAD mice, BAM2 cells showed reduced Aβ endocytosis, loss of mitochondrial membrane potential, increased mitochondrial and cytoplasmic reactive oxygen species, and greater senescence-associated activity. Experimental mitochondrial depolarization reduced Aβ uptake, linking metabolic competence directly to clearance capacity. Analyses of human AD tissue similarly suggested reduced oxidative-phosphorylation and reactive-oxygen-detoxification programs in BAM2-like cells, together with altered mitochondrial morphology. These findings support a self-reinforcing process in which Aβ uptake stresses BAM metabolism, metabolic dysfunction reduces further clearance, and reduced clearance increases extracellular and vascular Aβ exposure [40].

### 4.4. CD36 Converts Amyloid Recognition into Vascular Oxidative Stress

A central mechanism by which Aβ converts BAMs from clearance cells into vascular injury mediators involves CD36. CD36 is a class B scavenger and pattern-recognition receptor capable of recognizing fibrillar Aβ and other damage-associated ligands. Its activation can initiate signaling through NADPH oxidase complexes, particularly NOX2, resulting in reactive oxygen species production. At the neurovascular interface, this response is especially consequential because oxidative molecules produced by a BAM can act directly on adjacent endothelial cells, vascular smooth-muscle cells, pericytes, basement membranes, and vasoactive signaling pathways [11,27,28,67].

Initial genetic experiments showed that deletion of CD36 in Tg2576 mice selectively reduced vascular Aβ40 and CAA while preserving vascular LRP1 expression and improving cerebrovascular function. Parenchymal Aβ42-rich plaques were less affected, indicating a preferential role for CD36 in vascular rather than total amyloid pathology. The authors proposed that CD36 promotes CAA through a combination of oxidative vascular injury, impaired vasomotor activity, and reduced vascular Aβ clearance [67].

Park and colleagues subsequently localized an important component of this mechanism to perivascular macrophages. Selective depletion of these cells abolished ROS production and neurovascular dysfunction induced by Aβ applied to the cortex, administered intravascularly, or generated endogenously in Tg2576 mice. Replacement with bone marrow lacking CD36 or NOX2 similarly rescued neurovascular responses without requiring a reduction in total brain Aβ. These experiments identified perivascular macrophages as cellular intermediaries that translate Aβ recognition into vascular oxidative stress [27].

More recent lineage-directed work extended this pathway from acute neurovascular dysfunction to progressive CAA and cognitive impairment. Uekawa and colleagues replaced BAM compartments in Tg2576 mice with CD36-deficient or control cells. Loss of CD36 in BAMs suppressed macrophage ROS production, restored functional hyperemia and endothelial and smooth-muscle vasoreactivity, reduced vascular Aβ40 and CAA, and improved cognition. It did not substantially reduce parenchymal plaques or Aβ42. These findings provide strong evidence that the Aβ–CD36–NOX2 pathway within BAMs is not merely a reaction to established vascular pathology; it contributes causally to the retention of vascular Aβ and deterioration of neurovascular function [28].

The relationship between CD36 and Aβ is therefore paradoxical. A receptor involved in amyloid recognition and uptake can facilitate pathological clearance failure when its inflammatory and oxidative signaling outweighs its scavenging benefit. This may explain why broadly increasing macrophage activation is unlikely to be therapeutically sufficient. An effective BAM response would need to preserve uptake and degradation while limiting CD36-dependent oxidative signaling. Receptor expression, co-receptor availability, peptide aggregation state, metabolic reserve, and disease stage may determine which side of this balance predominates.

### 4.5. Aβ Alters the BAM Secretory Phenotype

BAM dysfunction also changes secretory output. In addition to soluble mediators and conventional extracellular vesicles, Aβ40 can induce TSPAN4-dependent macrophage migrasomes, providing a route for spatially focused signaling at the neurovascular interface.

Hu and colleagues (2023) showed that Aβ40 uptake increased production of CD5L/AIM-enriched migrasomes and that these structures could dock to vascular surfaces and promote complement-dependent barrier injury [57]. Because the human observations in that study were centered on CAA and on blood and peripheral skin rather than direct human brain-tissue demonstration, this pathway is best regarded as a vascular-amyloid mechanism requiring further human validation; its vascular actions are detailed in Section 6.

An unresolved question is whether APOE genotype modifies TSPAN4-dependent migrasome biogenesis. No published study identified in the present literature survey has directly compared APOE2-, APOE3-, and APOE4-expressing BAMs or macrophages for TSPAN4 induction, migrasome output, or CD5L/AIM cargo loading. Nevertheless, an interaction is biologically plausible. APOE is a major regulator of lipid transport and Aβ handling in CAA, and APOE4 can produce cell-autonomous BAM neurovascular dysfunction [17,72]. Conversely, TSPAN4-dependent migrasome biogenesis depends on lipid-organized tetraspanin macrodomains, membrane stabilization, sphingomyelin metabolism, and TSPAN4 palmitoylation [45,50,51,73]. APOE4-associated disturbances in BAM lipid handling and metabolic reserve could therefore, in principle, alter the membrane conditions required for Aβ40-triggered migrasome formation or the subsequent loading of CD5L/AIM as BAMs become metabolically exhausted. This remains a testable hypothesis rather than an established pathway and should be addressed directly in isogenic APOE2/3/4 BAM or macrophage models.

### 4.6. Chronic Aβ Exposure May Promote Senescent-like BAM States

Cellular senescence is a persistent stress-response state rather than a single-marker phenotype. Consensus frameworks emphasize convergent evidence across cell-cycle regulation, lysosomal and metabolic remodeling, macromolecular damage, resistance to apoptosis, and persistent secretory reprogramming, with no universally sufficient marker [74]. This requirement is particularly important for macrophages and microglia because many are slowly proliferating at baseline and can express p16INK4a or senescence-associated β-galactosidase (SA-β-gal) during reversible activation or polarization [75]. Accordingly, this review uses the term senescent-like BAM rather than definitively senescent BAM unless multiple orthogonal features support a durable state. Because many BAMs are long-lived and self-renew locally, they may accumulate damage during prolonged exposure to vascular Aβ, oxidized molecules, complement, and inflammatory signals.

Hu and colleagues (2025) [64] reported that BAMs acquired senescence-associated properties during relatively early brain aging. The authors proposed that prolonged Aβ exposure contributed to this state, although the study did not establish Aβ as the only initiating factor. Senescent-like BAMs produced increased numbers of migrasomes containing CD5L/AIM. These structures were taken up preferentially by microglia, where CD5L activated CD16-associated signaling, inhibited apoptosis, and promoted a secondary senescence-like phenotype. Suppression of TSPAN4-dependent migrasome production reduced microglial senescence and improved cognition in aged mice [64].

This result suggests a transition from cell-autonomous BAM damage to non-cell-autonomous propagation of dysfunction. Aβ and aging may initially stress BAMs at brain borders, but the resulting phenotype is not confined to those cells. Through migrasomes, BAMs can communicate senescence-associated signals to parenchymal microglia. Microglial resistance to apoptosis may appear protective in the short term, yet prolonged survival of damaged and dysfunctional cells could maintain inflammatory signaling, impair debris clearance, and interfere with tissue renewal. The subsequent BAM–migrasome–microglia pathway therefore offers a plausible mechanism through which chronic amyloid exposure at the vasculature can influence immune aging deeper within the brain.

Several uncertainties remain. The study investigated cognitive aging rather than a dedicated transgenic AD model, and its conclusion that Aβ contributes to BAM senescence was phrased cautiously. It remains unclear whether soluble Aβ, vascular fibrils, oxidative stress secondary to CD36 activation, or defective degradation is the dominant trigger. It is also unknown whether Aβ-induced migrasomes causing complement injury and senescent-BAM migrasomes transmitting paracrine senescence represent different stages of one pathway or separate responses occurring in distinct BAM populations.

### 4.7. Aβ May Impair BAM-Supported Perivascular Clearance

BAM dysfunction could increase amyloid burden through effects on the physical environment of perivascular spaces. Parenchymal border macrophages regulate extracellular-matrix composition and cerebrospinal-fluid flow dynamics. Changes in their number or phenotype can alter the resistance of perivascular pathways and the movement of fluid and solutes. Although this function has not yet been connected directly to the migrasome pathway in AD models, it provides a plausible feedback mechanism: Aβ changes BAM behavior; dysfunctional BAMs remodel the perivascular matrix or vessel wall; impaired perivascular transport then increases local Aβ retention and further BAM exposure.

CD36-dependent oxidative stress offers one route into this feedback loop. Reduced endothelial and smooth-muscle vasoreactivity diminishes pulsatile forces that may support perivascular fluid movement. Vascular injury can also alter basement-membrane composition, LRP1-dependent clearance, vessel stiffness, and extracellular-matrix organization. The observation that BAM-specific CD36 deletion restored vascular function and reduced Aβ40 suggests that improved amyloid disposal may arise partly from preservation of the vascular machinery required for clearance, rather than from direct macrophage degradation alone [27,28,67].

Aβ-related BAM dysfunction is therefore capable of producing a vicious cycle:

Vascular Aβ accumulation → BAM receptor activation and phagocytic loading → oxidative and metabolic stress → impaired neurovascular and clearance function → additional Aβ retention.

Migrasome release, complement activation, endothelial damage, and cellular senescence may become additional branches of this cycle. The relative importance of each branch is likely to vary between parenchymal-dominant AD, CAA-dominant disease, APOE genotypes, vascular comorbidities, and treatment states.

Recent evidence from APP knock-in mice places parenchymal border-macrophage loss and glymphatic dysfunction before extensive plaque deposition. Liu and colleagues reported impaired glymphatic influx and solute clearance in App^NL-F mice during an early disease stage, when substantial parenchymal plaque accumulation had not yet developed. The transport abnormality was closely associated with reduced numbers of parenchymal border macrophages and altered expression of macrophage-associated markers rather than with total plaque burden. Acute exposure of wild-type mice to CSF-delivered Aβ similarly reduced border-macrophage abundance and impaired glymphatic transport. Moreover, anti-Aβ antibody treatment reduced established amyloid pathology and partially restored border macrophages in selected regions but did not normalize glymphatic function. These findings suggest that soluble or CSF-borne Aβ may damage the macrophage component of the clearance system early and that removal of deposited plaques may be insufficient to reverse an established fluid-transport defect [76].

This sequence provides an additional potential feed-forward loop: soluble Aβ reduces parenchymal border-macrophage support, loss of these macrophages impairs CSF–interstitial transport, and reduced transport increases the residence time of soluble Aβ at brain borders. Migrasome production should be examined during this early stage, because quantitative loss of BAMs does not exclude increased migrasome release by the surviving, stressed population.

### 4.8. Anti-Aβ Antibodies Create an Additional Form of BAM Exposure

Therapeutic mobilization of Aβ changes both the amount and immunological form of amyloid encountered by BAMs. Antibody-bound vascular Aβ forms immune complexes that can activate macrophage Fc receptors, increasing phagocytosis but also inflammatory signaling and extracellular-matrix remodeling. In aged PDAPP mice, treatment with a murine analogue of bapineuzumab caused antibody binding to vascular amyloid, activation of CD169-positive perivascular macrophages, increased expression of inflammatory and matrix-remodeling genes such as Timp1 and Mmp9, plasma-protein extravasation, and recruitment of inflammatory monocytes around vascular deposits and microhemorrhages [29].

Subsequent spatial analyses linked Aβ immunotherapy-associated vascular injury with smooth-muscle-cell loss, blood–brain barrier breakdown, vascular fibrosis, and both resident and monocyte-derived TREM2-positive macrophage populations around CAA. These studies do not prove that BAM activation is the sole cause of amyloid-related imaging abnormalities, but they demonstrate that Aβ–antibody complexes can reprogram the perivascular immune niche and that resident BAMs participate in the response to therapeutic amyloid mobilization [77].

This has two implications. First, BAM functional reserve may influence whether mobilized Aβ is removed safely or redistributed toward vulnerable vessels. Second, the same macrophage can potentially receive competing signals: scavenger-receptor stimulation by Aβ, Fc-receptor activation by antibody–Aβ complexes, complement-receptor engagement, oxidative stress, and matrix-derived signals from an injured vessel. Therapeutic interventions directed at BAMs must therefore account for both spontaneous disease and the altered immune environment created by anti-Aβ treatment.

### 4.9. Human Evidence and Unresolved Questions

Direct demonstration of Aβ-driven BAM dysfunction in living humans remains limited. Human leptomeningeal single-cell studies show that BAM populations in AD exhibit altered complement, extracellular-matrix, APP-related, antigen-presentation, SPP1, and TGF-β-associated programs. These observations establish that the human border immune niche is remodeled in AD but cannot determine whether Aβ directly initiated each transcriptional change. Postmortem analysis also cannot easily distinguish long-lived resident BAMs from recruited monocyte-derived macrophages or activated microglia without combined spatial and molecular evidence [6].

Human macrophage experiments suggest impaired Aβ clearance and increased apoptosis in AD, while the CAA cohort provides human-associated evidence of increased macrophage-lineage migrasome production and complement activation. The 2026 BAM2 bioRxiv preprint further reports reduced BAM abundance and metabolic capacity in human AD datasets and tissue imaging. These findings are directionally consistent with progressive BAM exhaustion, but their evidence levels differ: the CAA observations are peer-reviewed but largely peripheral, whereas the BAM2 human analyses remain preprint data. Neither establishes a longitudinal human sequence from Aβ exposure to BAM oxidative/metabolic failure, senescence-like reprogramming, migrasome release, and cognitive decline [40,57,66].

Several variables require systematic investigation. It is not known whether BAM dysfunction begins during preclinical amyloidosis or only after substantial CAA has developed. The effects of Aβ40 and Aβ42 should be compared directly across BAM subsets, aggregation states, and anatomical niches. APOE genotype, sex, vascular risk, sleep, systemic inflammation, and age may influence BAM lipid metabolism and degradative reserve. The relationship between CD36-mediated oxidative signaling and SR-BI-associated protective responses remains incompletely resolved. Similarly, the mechanisms that sort CD5L and other proteins into Aβ-induced migrasomes are unknown.

The concept of Aβ-driven BAM dysfunction should therefore be framed as a dynamic trajectory. Functional BAMs initially support amyloid uptake, vascular surveillance, and perivascular homeostasis. Persistent Aβ exposure can progressively compromise these functions through phagolysosomal overload, CD36–NOX2-dependent oxidative stress, mitochondrial exhaustion, altered extracellular-matrix interactions, complement-amplifying migrasomes, and senescence-associated signaling. The resulting cells may no longer restrict vascular amyloid and may instead contribute to CAA, blood–brain barrier damage, neurovascular uncoupling, microglial dysfunction, and cognitive decline. This trajectory provides the biological foundation for the proposed BAM–migrasome–microglia senescence axis, while emphasizing that therapeutic strategies must preserve beneficial BAM clearance rather than suppressing the entire population.

BAM function may also influence tau pathology: depletion of parenchymal border macrophages aggravated tau pathology and neurodegeneration in PS19 mice. This finding supports the importance of preserving beneficial BAM functions, but no study has shown that tau induces migrasomes or that BAM-derived migrasomes directly alter tau uptake or seeding [78].

## 5. The BAM–Migrasome–Microglia Senescence Axis

Cellular senescence is increasingly implicated in brain aging and AD, but senescence within the central nervous system should not be understood solely as the independent accumulation of damaged cells. Senescent and senescent-like cells can modify surrounding tissues through cytokines, chemokines, growth factors, lipids, extracellular vesicles, and other components of the senescence-associated secretory phenotype. The recent identification of migrasome-mediated signaling between BAMs and microglia extends this framework by suggesting that senescence can be transmitted from brain-border compartments into the parenchyma through a spatially organized extracellular organelle system. This proposed BAM–migrasome–microglia senescence axis provides a mechanistic link among aging, vascular Aβ exposure, macrophage dysfunction, altered cell survival, microglial senescence, and cognitive deterioration [64].

The central sequence can be summarized as follows:

Aging and persistent Aβ40 uptake → senescent-like BAM reprogramming → increased TSPAN4-dependent migrasome production → enrichment and transfer of CD5L/AIM → activation of CD16-associated signaling in microglia → inhibition of apoptosis and persistence of damaged microglia → secondary microglial senescence, inflammatory signaling, and impaired tissue clearance.

This model is biologically attractive because BAMs occupy the perivascular and meningeal routes through which Aβ-containing interstitial and cerebrospinal fluids are cleared. Migrasomes produced at these interfaces can remain associated with extracellular-matrix and vascular structures or move from perivascular spaces into the adjacent brain. Microglia therefore need not contact the original senescent-like BAM directly: the pathological message can persist after the donor macrophage has moved away. Nevertheless, direct experimental support for the complete pathway currently rests predominantly on one extensive 2025 study of physiological mouse aging, supplemented by cell-culture experiments and related work on Aβ-induced macrophage migrasomes and microglial senescence. The framework should consequently be presented as an emerging mechanistic axis rather than an established pathway in human AD [57,64].

### 5.1. BAMs May Be Early Initiators of Brain-Cell Senescence

A defining feature of the proposed axis is its directionality. BAMs are not interpreted merely as one of several cell populations becoming dysfunctional during advanced aging; they may represent an early site from which senescence-associated signals spread to other brain cells. Hu and colleagues (2025) [64] integrated newly generated and publicly available single-cell RNA-sequencing datasets from male and female mice across several ages. BAMs showed relatively early enrichment of the SenMayo senescence-associated gene set and increased expression of senescence-associated secretory mediators. In parallel experiments, CD206-positive BAMs displayed age-related expression of TNF-α and IFN-γ, whereas overt *Cdkn2a*-associated changes in neurons, astrocytes, oligodendrocytes, and microglia were detected later. These observations led the authors to propose that BAMs are among the leading cellular populations entering a senescence-associated state during brain aging [64].

The designation senescent-like BAM is appropriate because macrophage senescence cannot be defined by a single marker. Operationally, a strong assignment should combine evidence from several domains: (i) sustained reduction in proliferative competence or altered cell-cycle control, assessed with Ki67/EdU together with p16INK4a-, p21- or RB-pathway changes where biologically interpretable; (ii) lysosomal remodeling, including SA-β-gal activity and/or lipofuscin accumulation; (iii) persistent macromolecular-damage features such as γH2AX/DNA-damage signaling, telomere abnormalities, loss or redistribution of lamin B1, or HMGB1 relocalization; (iv) mitochondrial or metabolic dysfunction; (v) resistance to apoptosis; and (vi) a persistent SASP-like inflammatory/secretory program [74]. Ideally, persistence after withdrawal of the initiating stimulus or failure to return to a baseline functional state should also be demonstrated. This multi-domain requirement is essential because p16INK4a and SA-β-gal can be induced reversibly in macrophages by physiological stimuli and therefore cannot, by themselves, distinguish senescence from activation [75]. The 2025 study used a convergent panel including Cdkn2a and p21-related signaling, SA-β-gal, cellular and nuclear enlargement, lipofuscin, DNA-damage-associated markers, altered lamin B1 and HMGB1 localization, telomere changes, inflammatory mediators, and apoptosis resistance. The combined pattern supports senescence-associated reprogramming, but the term senescent-like remains the most rigorous description [64].

The anatomical position of BAMs may explain why this state emerges early. Perivascular and leptomeningeal macrophages continuously encounter molecules leaving the brain, including Aβ, myelin fragments, APOE-containing particles, oxidized lipids, and other products of neural and vascular metabolism. In the 2025 study, leptomeningeal BAMs contained several brain-derived substances, and intracisternal delivery experiments indicated that CD206-positive BAMs efficiently engulfed Aβ40. Such repeated uptake may initially support clearance but can eventually impose lysosomal, oxidative, and mitochondrial stress. Because BAMs are relatively long-lived, incompletely degraded material may accumulate over time and progressively alter their survival, secretion, and migratory behavior [64].

### 5.2. Aβ40 Connects Brain-Border Exposure with Senescent-like BAM Reprogramming

The upstream role of Aβ was investigated most directly using Aβ40, the peptide species particularly relevant to vascular and perivascular amyloid deposition. Age-related Aβ40 accumulation was detected in BAMs, while exposure of mouse bone-marrow-derived macrophages to Aβ40 induced a broad senescence-associated phenotype. Treated macrophages showed altered cell-cycle distribution, cellular enlargement, increased senescence-associated β-galactosidase activity, telomere shortening, lipofuscin accumulation, DNA-damage-associated changes, and altered expression of p21, lamin B1, HMGB1, phosphorylated STAT3, and other senescence-related markers. Similar changes were reproduced in RAW264.7 cells and human monocyte-derived macrophages, indicating that the response was not confined to one murine cell preparation [64].

These experiments establish that Aβ40 is sufficient to induce a senescence-associated macrophage phenotype under the tested conditions, but they do not establish that Aβ is the sole physiological trigger of BAM senescence. Aging also exposes brain-border macrophages to vascular stiffness, disturbed cerebrospinal-fluid flow, systemic inflammatory mediators, mitochondrial damage, extracellular-matrix remodeling, and altered lipid metabolism. Aβ may therefore operate as one component of a cumulative stress environment. The phrase used in the original report—senescence occurring “possibly due to prolonged exposure to amyloid beta”—appropriately reflects this uncertainty [64].

Independent studies nevertheless strengthen the general connection between Aβ exposure and myeloid-cell senescence. In human HMC3 microglial cells, Aβ increased p21, PAI-1, senescence-associated β-galactosidase activity, inflammatory cytokine expression, mitochondrial reactive oxygen species, and loss of respiratory and membrane-potential function. These changes were associated with suppression of the SIRT1–NRF2 pathway and impaired phagocytosis, while restoration of SIRT1 counteracted several abnormalities [79]. In an unrelated study, Y. Hu and colleagues (2022) found in BV2 cells and APP/PS1 mice that Aβ40-associated senescence was linked to increased CD38 activity, depletion of NAD^+^-related metabolic capacity, increased inflammatory cytokines, and cognitive impairment; CD38 inhibition partly reversed these changes [80].

Aβ-driven BAM senescence and Aβ-driven microglial senescence may thus represent two connected but distinguishable processes. Microglia can become senescent through direct contact with parenchymal Aβ, especially when autophagic and metabolic defenses fail. The BAM–migrasome pathway adds an indirect route in which Aβ is first sensed and internalized at the border, reprogramming a macrophage that subsequently transmits a secondary senescence-inducing signal into the brain. These pathways may act simultaneously and reinforce one another rather than competing as alternative explanations.

### 5.3. Senescent-like BAMs Switch from Soluble Secretion to Migrasome-Mediated Signaling

Senescent-like BAMs were found to increase their production of TSPAN4-positive migrasomes with age. This is more than a quantitative increase in generic extracellular-vesicle release. Migrasomes form on retraction fibers generated during macrophage migration and can therefore be deposited along perivascular and meningeal extracellular-matrix routes. Age-dependent increases in BAM TSPAN4 expression and in CD206-positive migrasomal structures suggest that aging alters not only the molecular content of BAM secretion but also its spatial organization [64].

Migrasomes isolated from aged mouse brains were enriched for structures derived from CD206-positive BAMs. After fluorescent labeling and intracisternal administration, BAM-derived migrasomes could move from perivascular spaces toward the brain parenchyma. Migrasomes from aged mice also altered cell-cycle and senescence-associated measures in organotypic brain-slice cultures. These experiments provide a physical bridge between border macrophages and parenchymal cells: a BAM-generated organelle is capable of crossing the immediate border niche and reaching potential recipient populations inside the brain [64].

The study additionally compared migrasomes with exosomes and soluble fractions released by Aβ40-exposed macrophages. Aβ40-induced migrasomes were sufficient to induce lipofuscin accumulation, senescence-associated β-galactosidase activity, cell-cycle changes, telomere abnormalities, p21 expression, and inflammatory mediators in cultured microglia. The experiments identified the migrasomal fraction as a major carrier of the senescence-inducing activity, distinguishing the pathway from conventional diffusion of soluble cytokines or generalized extracellular-vesicle release [64].

This does not mean that migrasomes replace the conventional senescence-associated secretory phenotype. Senescent-like BAMs can presumably produce soluble cytokines and other extracellular vesicles at the same time. Migrasomes instead add several properties: concentrated cargo loading, protection of cargo within a membrane structure, adhesion to vascular or extracellular-matrix surfaces, deposition along the donor cell’s migration route, and delayed delivery after the BAM has left the original site. They may consequently extend the spatial range and duration of BAM-derived senescence signals.

### 5.4. Microglia Are Prominent Recipients of BAM-Derived Migrasomes

Microglia were identified as prominent recipient cells for senescent-like BAM-derived migrasomes. Their susceptibility is anatomically plausible because microglial processes survey the parenchyma near penetrating vessels and can internalize extracellular particles reaching the brain through perivascular routes. Microglia are also highly phagocytic, making uptake of large membrane-bound structures more likely than in many neuronal populations. The transfer experiments indicated that migrasomes released at brain borders can gain access to microglia without requiring entry of the entire BAM into the parenchyma [64].

Recipient specificity is not yet fully understood. Microglia may be preferentially exposed because of their proximity to the perivascular space, their capacity for particle engulfment, or the expression of receptors recognizing migrasomal membrane proteins and cargo. It is also possible that other cells receive migrasomes but respond differently. Astrocytes, endothelial cells, pericytes, neurons, and oligodendroglial cells were not established as equivalent recipients in the central mechanistic experiments. Future studies will need to determine whether recipient selection is governed by migrasome adhesion, phagocytosis, receptor expression, local extracellular matrix, or disease-dependent changes in vascular permeability.

Microglial uptake did not produce an immediate cytotoxic response. Instead, the recipient cells exhibited reduced proliferation or altered cell-cycle distribution, increased p21, senescence-associated β-galactosidase activity, lipofuscin accumulation, shortened telomeres, and greater TNF-α and IFN-γ expression. This phenotype supports a transition toward persistent dysfunctional survival rather than acute cell death. In the context of AD, prolonged survival of damaged microglia may be particularly detrimental because it preserves cells with reduced clearance capacity and sustained inflammatory output.

### 5.5. CD5L/AIM Is a Central Senescence-Regulatory Migrasomal Cargo

Proteomic and mechanistic analyses identified apoptosis inhibitor of macrophage—AIM, also known as CD5 antigen-like protein or CD5L—as a major functional cargo of senescent-like BAM-derived migrasomes. CD5L/AIM is a secreted scavenger-receptor-cysteine-rich protein produced predominantly by macrophage-lineage cells. It was originally identified as a factor capable of increasing cell survival and inhibiting apoptosis, and subsequent studies showed that it can preserve lipid-loaded macrophages in chronically inflamed tissues [81,82].

In the BAM–microglia experiments, Aβ40 stimulation increased AIM-containing migrasomes. Purified AIM was itself sufficient to induce several senescence-associated changes in brain-slice or microglial cultures. More importantly, migrasomes generated by *Cd5l*-deficient macrophages had a substantially reduced ability to induce microglial senescence. The effect therefore depended not merely on the migrasomal membrane or particle uptake but on a specific donor-derived cargo [64].

AIM should not be classified as an intrinsically neurotoxic molecule. In experimental ischemic stroke, AIM bound damage-associated molecular patterns, promoted their phagocytic removal, limited sterile inflammation, and improved survival after recombinant administration [83]. More recently, CD5L/AIM was shown to bind Aβ oligomers, reduce their aggregation, promote microglial uptake, reduce plaque burden, and improve cognition in 5xFAD mice [84]. Its biological effect is therefore strongly context-dependent rather than intrinsically pathological. The outcome may depend on molecular form, carrier state, concentration, receptor availability, tissue compartment, co-cargo, duration of exposure, and whether increased cell survival enables functional recovery or instead prevents elimination of irreversibly damaged cells.

A second distinction is required between systemic soluble CD5L/AIM and locally concentrated migrasome-associated exposure. Circulating CD5L is not simply a freely diffusible monomeric pool: structural work shows that CD5L associates with J-chain-containing pentameric IgM, including a Ca2+-dependent J-chain interaction and disulfide linkage [85]. Thus, plasma concentration reflects a carrier-associated systemic pool and cannot be assumed to equal the concentration, molecular accessibility, or receptor occupancy achieved at a tissue surface. In the CAA migrasome experiments, soluble CD5L at circulating concentrations was insufficient to reproduce endothelial injury, whereas intact, vessel-docking migrasomes generated a highly concentrated local CD5L microdomain and produced barrier toxicity [57]. The mechanistic claim in this review therefore concerns spatially concentrated, migrasome-associated CD5L exposure under defined vascular or recipient-cell conditions, not a claim that higher systemic CD5L is intrinsically harmful.

This duality is central to the proposed axis. Apoptosis is not invariably harmful in aging tissues: controlled death and replacement of severely damaged immune cells can prevent their long-term accumulation. AIM-mediated survival may initially protect microglia during stress, but persistent inhibition of apoptosis could allow dysfunctional cells to remain within the parenchyma. Senescence may consequently emerge as the alternative fate of microglia that receive survival signals despite accumulating metabolic, lysosomal, DNA, or oxidative damage.

### 5.6. CD16-Associated Signaling Links AIM Delivery to Apoptosis Resistance

Hu and colleagues (2025) [64] implicated a recipient-side CD16/Fcγ-receptor mechanism. Microglial Fcgr3 expression increased with age, Aβ40-induced migrasomes increased CD16 protein/signaling readouts in microglial and organotypic-slice cultures, and Fcγ-receptor blockade attenuated migrasome-induced senescence markers and lipofuscin accumulation. Loss of AIM from donor-cell migrasomes similarly weakened the response [64]. These experiments establish functional dependence on an Fcγ-receptor-sensitive recipient pathway and are consistent with a role for Fcgr3/CD16, but they do not by themselves demonstrate a direct CD5L–CD16 ligand–receptor interaction.

Receptor-level interpretation requires additional caution. FcγRIII/CD16 is classically a low-affinity IgG receptor whose activating functions depend on associated FcRγ-chain immunoreceptor tyrosine-based activation motifs and downstream Src/Syk-centered signaling, with context-dependent engagement of PI3K–AKT, MAPK, phagocytic, and inflammatory programs [86]. Anti-apoptotic signaling is therefore not an invariant intrinsic function of CD16; the observed apoptosis resistance is an outcome of this experimental context. Moreover, the neutralizing antibody used in the 2025 study was clone 2.4G2, a reagent known to recognize murine FcγRIII/CD16 and FcγRII/CD32 [87]. The blockade experiment should consequently be interpreted as FcγRII/III-sensitive rather than CD16-exclusive evidence. Direct physical binding between CD5L/AIM and CD16 has not yet been demonstrated, and other migrasomal components, receptor co-complexes, or membrane organization could contribute to receptor activation. Definitive assignment will require Fcgr3-specific genetic loss-of-function or rescue, separation from FcγRII effects, direct binding assays using purified proteins or cell-surface crosslinking, and mapping of FcRγ/Syk and downstream survival effectors such as AKT, BCL-2-family proteins, and caspases. Until these experiments are performed, the most accurate wording is ‘CD5L/AIM-dependent, CD16-associated Fcγ-receptor signaling’ rather than direct ‘CD5L binding to CD16.’

Important species differences further limit direct translation of the CD5L/AIM–CD16 mechanism from mouse to human. In mice, FcγRIII is encoded by *Fcgr3*, whereas humans express two distinct CD16 receptors, FCGR3A (CD16A) and FCGR3B (CD16B), which differ in cellular distribution, membrane anchoring, glycosylation, and signaling capacity. CD16A is a transmembrane receptor expressed predominantly on natural killer cells and subsets of monocytes/macrophages and signals through associated FcRγ or CD3ζ chains, whereas CD16B is a glycosylphosphatidylinositol-anchored receptor expressed mainly on neutrophils and lacks a direct murine equivalent. These differences are particularly relevant when extrapolating macrophage or microglial findings to human disease, because receptor availability and downstream signaling cannot be assumed to be identical across species. In addition, circulating human CD5L/AIM is largely associated with IgM, which influences its stability, distribution, and tissue availability and may differ from the locally concentrated CD5L/AIM presented within migrasomes. Therefore, the biological consequences of migrasome-associated CD5L/AIM in human brain-border macrophages and microglia require direct validation rather than inference from murine Fcgr3-dependent experiments.

### 5.7. Senescent Microglia Can Amplify Alzheimer’s Disease Pathology

The pathological importance of the axis depends on whether the induced microglial state meaningfully affects AD progression. Independent studies indicate that senescent or senescence-associated microglia have reduced proliferative and phagocytic competence, dystrophic morphology, metabolic dysfunction, and sustained inflammatory secretion. Aβ-induced human microglial senescence was accompanied by mitochondrial impairment and reduced phagocytosis through suppression of SIRT1–NRF2 signaling [79]. Separately, Y. Hu and colleagues (2022) linked Aβ40-induced CD38 expression to NAD^+^ depletion, energetic failure, and increased inflammatory cytokines, while CD38 inhibition improved metabolic and cognitive outcomes in APP/PS1 mice [80].

Microglial autophagy appears to oppose this trajectory. Choi and colleagues found that autophagy was activated in disease-associated microglia surrounding amyloid plaques. Conditional disruption of microglial autophagy caused microglial disengagement from plaques, reduced the disease-associated response, increased p21-positive and dystrophic senescence-associated microglia, and aggravated AD-like neuropathology. Pharmacological elimination of autophagy-deficient senescent microglia alleviated pathology, suggesting that persistence of these cells was functionally harmful rather than merely a marker of advanced disease [88].

The BAM–migrasome pathway may therefore operate against an important microglial protective mechanism. Plaque-associated microglia require autophagy, lysosomal function, mitochondrial energy, and receptor-mediated signaling to maintain plaque containment and debris clearance. Repeated AIM–CD16-mediated survival signaling could retain microglia whose autophagic or metabolic systems are already compromised. These cells may remain alive but lose the ability to compact plaques, remove damaged myelin, clear dystrophic neurites, or resolve inflammatory responses.

Senescent microglia could also contribute to a self-reinforcing feedback loop. Reduced Aβ clearance increases extracellular and vascular amyloid burden; greater Aβ exposure further stresses BAMs and induces additional migrasome production; AIM-rich migrasomes then generate more senescent-like microglia. Inflammatory mediators released by recipient microglia may additionally alter endothelial cells, astrocytes, perivascular extracellular matrix, and other BAMs. Thus, an initially localized signal at a brain border could be converted into a multicellular inflammatory field extending through the neurovascular unit and parenchyma.

The consequences may extend beyond amyloid. In PS19 tauopathy mice, p16-positive microglia and astrocytes accumulated before severe neurodegeneration. Genetic clearance of these cells prevented gliosis, tau hyperphosphorylation and aggregation, neuronal loss, and cognitive decline, while senolytic treatment altered tau pathology [89]. This does not prove that BAM-derived migrasomes cause tau-associated senescence, but it supports the possibility that microglial senescence initiated during amyloid and vascular disease could later influence tau propagation and neuronal degeneration [89].

A potential second-stage amplifier of migrasome-induced microglial dysfunction is the integrated stress response. Flury and colleagues identified an integrated-stress-response-active microglial state associated with the ultrastructural characteristics of dark microglia, synaptic injury, and adverse neurodegenerative outcomes. Experimental activation of this pathway in microglia aggravated AD-related pathology and synapse loss, whereas inhibition of the stress response produced protective effects. Integrated stress signaling also stimulated the production and extracellular release of neurotoxic lipids, and inhibition of either the stress pathway or downstream lipid synthesis reduced synaptic damage. These findings demonstrate that chronically stressed microglia can become actively neurotoxic through metabolic and lipid-secretory mechanisms rather than solely through conventional pro-inflammatory cytokines [90].

This mechanism could operate downstream of the proposed BAM–migrasome pathway. CD5L/AIM-mediated inhibition of microglial apoptosis may permit damaged microglia to persist, while chronic CD16-associated signaling, mitochondrial dysfunction, impaired proteostasis, and amyloid exposure could activate the integrated stress response. The surviving cells might then release toxic lipids and promote synaptic loss. This connection remains hypothetical, because a direct causal relationship between migrasomal CD5L/AIM, CD16 signaling, and the microglial integrated stress response has not yet been tested.

### 5.8. Relationship with the Vascular Migrasome–Complement Pathway

The BAM–microglia senescence pathway may coexist with a second migrasome-mediated mechanism at the neurovascular interface. In experimental cerebral amyloid angiopathy, Aβ40 stimulated macrophage-lineage cells to release CD5L/AIM-enriched migrasomes that adhered to vascular surfaces, reduced the complement inhibitor CD59, increased C5b–9 membrane-attack-complex deposition, and damaged the blood–brain barrier [57].

The two mechanisms share an Aβ40-exposed macrophage donor and AIM-rich migrasomal cargo but differ in the principal recipient and outcome. In the vascular mechanism, migrasomes act on endothelial and complement-regulatory systems, producing barrier damage. In the senescence mechanism, migrasomes reach microglia and activate CD16-associated anti-apoptotic signaling. These pathways may represent different consequences of the same macrophage secretory reprogramming, different migrasome subtypes, or stage-specific responses. Early vascular deposition could promote complement injury, whereas prolonged aging and repeated migrasome exposure could extend the signal into the parenchyma and induce microglial senescence.

A combined model is therefore plausible:

Aβ40 uptake by BAMs → increased AIM-rich migrasomes → vascular deposition and complement injury + parenchymal uptake and microglial senescence.

Blood–brain barrier disruption caused by the first branch could further increase the accessibility of migrasomes or inflammatory molecules to parenchymal cells, thereby strengthening the second branch. However, the two outcomes have not yet been demonstrated concurrently in the same AD model, and their temporal relationship remains unknown.

**Figure 2:**
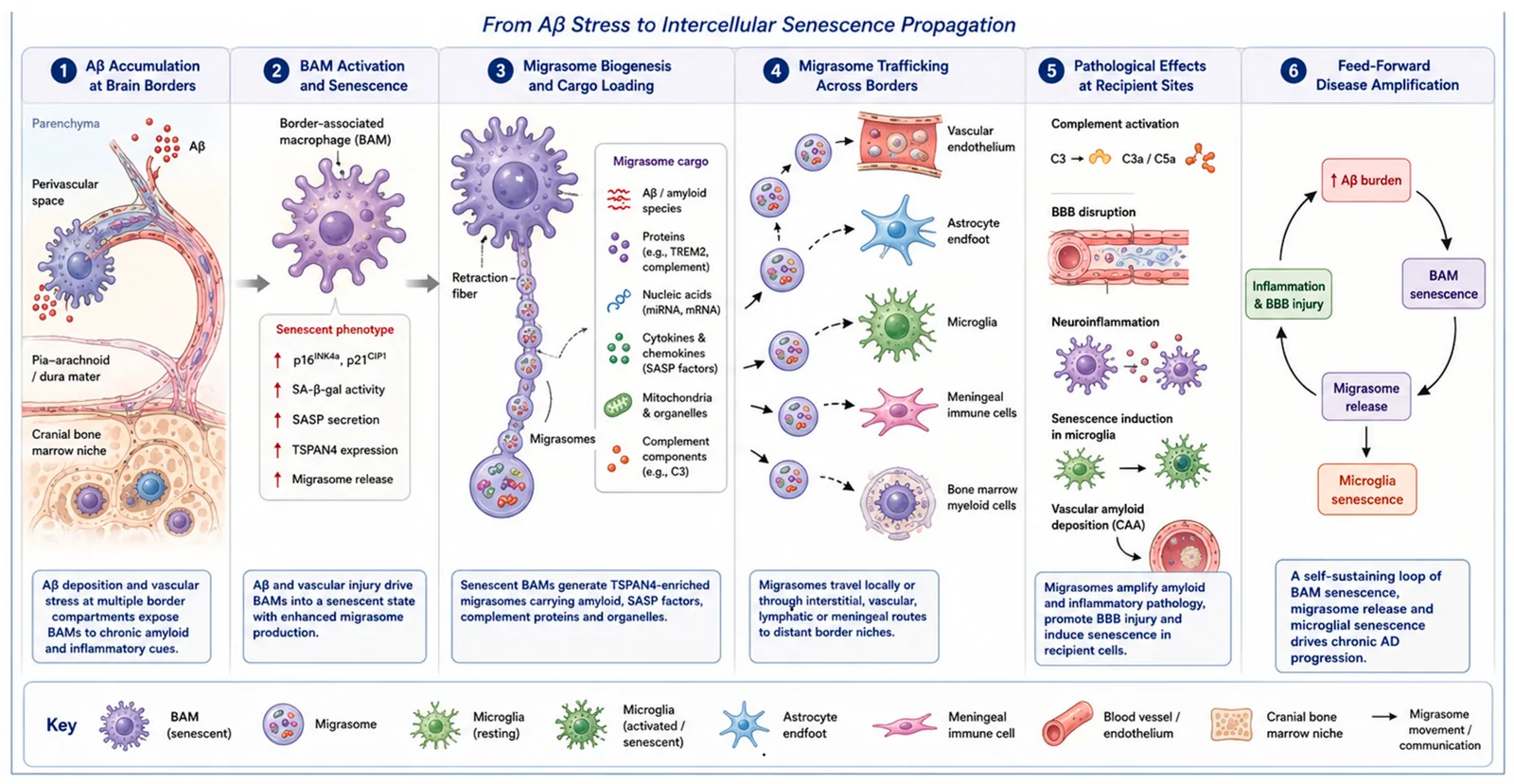
Proposed border-associated macrophage–migrasome–microglia senescence axis in Alzheimer’s disease. (1) Amyloid-β, particularly vascular Aβ40, accumulates along perivascular and meningeal clearance routes, exposing border-associated macrophages to persistent amyloid, oxidative, vascular, and inflammatory stress. (2) Chronic exposure may progressively shift functionally competent BAMs toward metabolically impaired or senescent-like states characterized by increased senescence-associated signaling and enhanced TSPAN4-dependent migrasome production. (3) Migrasomes form on retraction fibers and may contain CD5L/AIM, proteins, nucleic acids, cytokines, chemokines, complement-related molecules, and damaged organelles. The precise cargo composition is likely to depend on the BAM subset, anatomical niche, metabolic state, and disease stage. (4) Following release, migrasomes may remain associated with perivascular extracellular matrix, dock to vascular surfaces, or be taken up by nearby recipient cells. Direct trafficking to all illustrated brain-border compartments has not yet been demonstrated and should be interpreted as a proposed communication framework. (5) At vascular sites, Aβ40-induced CD5L/AIM-rich migrasomes may reduce endothelial complement resistance, promote C5b–9 membrane-attack-complex deposition, disrupt tight-junction integrity, and contribute to blood–brain barrier leakage and white-matter injury. In the parenchymal branch, BAM-derived migrasomes may be internalized by microglia, where CD5L/AIM–CD16-associated signaling inhibits apoptosis and promotes the persistence of damaged, senescent-like microglia. These cells may exhibit metabolic dysfunction, impaired phagocytosis, and increased inflammatory signaling. (6) Reduced amyloid clearance, vascular injury, microglial dysfunction, and chronic inflammation may subsequently increase Aβ retention and further stimulate BAM dysfunction and migrasome release, establishing a self-amplifying pathological cycle. Solid arrows denote relationships supported directly by experimental evidence, whereas dashed arrows denote proposed recipient cells, inter-compartmental routes, or mechanistic connections that remain incompletely validated in Alzheimer’s disease.

The proposed sequence linking Aβ exposure, BAM dysfunction, migrasome production, and secondary injury is summarized in Figure 2. Persistent exposure to vascular Aβ and aging-related stress may induce a senescent-like BAM phenotype characterized by increased TSPAN4-dependent migrasome formation and altered cargo loading. These migrasomes may then produce two partially overlapping pathological outcomes: vascular deposition with complement-mediated blood–brain barrier injury and transfer of CD5L/AIM-associated survival signals to microglia, promoting the persistence and senescence-like reprogramming of dysfunctional cells. The resulting impairment of amyloid clearance, neurovascular integrity, and immune homeostasis may establish a feed-forward cycle that further increases Aβ burden and chronic neuroinflammation.

## 6. Migrasomes in Cerebral Amyloid Angiopathy and Blood–Brain Barrier Injury

Cerebral amyloid angiopathy (CAA) is characterized by amyloid-β deposition within cortical and leptomeningeal vessel walls, where Aβ40 is generally more prominent than the relatively plaque-enriched Aβ42 species. Vascular amyloid is accompanied by progressive degeneration of vascular smooth-muscle cells, basement-membrane remodeling, impaired vasoreactivity, blood–brain barrier (BBB) leakage, microinfarction, cortical superficial siderosis, cerebral microbleeds, and lobar intracerebral hemorrhage. These abnormalities have historically been attributed to the direct toxicity and mechanical effects of vascular amyloid, impaired perivascular clearance, oxidative stress, and local inflammation. The identification of Aβ40-induced macrophage-derived migrasomes adds another mechanism: amyloid-exposed myeloid cells may deposit adhesive, cargo-rich structures on the vascular wall, thereby transforming a transient macrophage response into spatially concentrated and persistent complement-mediated endothelial injury [27,28,57,91,92,93,94,95,96].

The proposed pathway can be summarized as follows:

Vascular Aβ40 uptake by macrophage-lineage cells → TSPAN4-dependent migrasome production → vascular docking of CD5L/AIM-rich migrasomes → reduction in endothelial complement resistance → C5b–9 membrane-attack-complex formation → tight-junction loss, endothelial injury, BBB leakage, white-matter damage, and amplification of CAA.

This model is supported most directly by Hu and colleagues (2023), who combined macrophage cultures, endothelial barrier systems, migrasome-transfer experiments, two amyloid mouse models, and observations from a small human CAA cohort [57]. Other human neuropathological and imaging studies independently confirm that BBB disruption and complement activation occur in CAA, but they do not yet establish that migrasomes are the dominant cause of these abnormalities in patients [27,91,92,93,94,95,96].

### 6.1. Blood–Brain Barrier Injury Is an Early and Progressive Feature of CAA

The BBB is maintained by endothelial tight junctions, low endothelial transcytosis, pericytes, vascular smooth-muscle cells, basement membranes, astrocytic endfeet, and perivascular immune cells. In CAA, Aβ accumulates within the same vascular and basement-membrane structures that support solute clearance and maintain barrier integrity. Vascular amyloid can therefore impair the BBB through several overlapping mechanisms: direct endothelial toxicity, oxidative stress, loss or redistribution of tight-junction proteins, matrix-metalloproteinase activation, smooth-muscle-cell degeneration, basement-membrane disorganization, complement activation, and inflammatory signaling from perivascular cells [27,91,92,93,94,95,96].

Hartz and colleagues examined isolated brain microvessels from Tg2576 mice, rat microvessels exposed to Aβ, and human CAA tissue. Aβ exposure altered tight-junction and matrix-metalloproteinase expression, while the mouse and human material showed evidence of reduced barrier integrity. The findings supported a causal role for Aβ in disrupting microvascular junctional organization rather than treating leakage as merely a consequence of advanced hemorrhagic vessel destruction [91].

Magaki and colleagues subsequently examined human AD brains with capillary and non-capillary forms of CAA. Using endothelial, basement-membrane, tight-junction, and leakage markers, they identified alterations in CD31, collagen IV, claudin-5, and fibrinogen that varied according to the anatomical pattern of vascular amyloid. Their results reinforced the view that CAA affects multiple elements of the neurovascular unit and that capillary amyloid is particularly relevant to BBB disruption [92].

Postmortem MRI–histopathology studies have further shown that BBB leakage is associated with CAA-related microvascular lesions. Freeze and colleagues detected fibrinogen leakage around CAA-affected vessels and linked barrier abnormalities with microbleeds and microinfarcts [93]. Kozberg and colleagues then examined individual arterioles across progressive stages of CAA pathology. Fibrinogen accumulation and smooth-muscle-actin loss were already more frequent in vessels with mild Aβ deposition than in amyloid-negative vessels, while every vessel showing advanced remodeling displayed BBB leakage. Reactive astrocytes and activated myeloid cells were more prominent at later pathological stages, suggesting that leakage may precede and subsequently recruit perivascular inflammation [94].

The existence of BBB leakage in living patients was supported by an exploratory 2025 contrast-enhanced MRI study. Fourteen patients with probable CAA without previous intracerebral hemorrhage and seven non-CAA participants with cognitive complaints or mild cognitive impairment underwent post-contrast FLAIR and dynamic contrast-enhanced imaging. Sulcal cerebrospinal-fluid enhancement was observed only in the CAA group and was associated with greater cortical superficial siderosis volume. Cortical small-vessel permeability was numerically higher in CAA, although the between-group difference did not reach conventional statistical significance. These results suggest that leptomeningeal and possibly cortical BBB leakage may be measurable before a major hemorrhage, but the cohort was small and the findings require longitudinal replication [95].

These observations also provide a rationale for longitudinal multimodal imaging rather than relying on static hemorrhagic markers alone. DCE-MRI and post-contrast FLAIR can quantify parenchymal and leptomeningeal leakage, whereas arterial-spin-labeling and BOLD-fMRI with task or hypercapnic cerebrovascular-reactivity paradigms can capture perfusion reserve and neurovascular uncoupling; reduced whole-brain cerebrovascular reactivity has already been demonstrated in CAA, and recent fMRI work suggests that early CAA-related neurovascular impairment may be accompanied by localized functional-connectivity abnormalities [95,97,98,99]. Serial high-resolution structural, susceptibility, and diffusion/perivascular-space measures could additionally serve as indirect in vivo surrogates of vessel-wall and perivascular matrix remodeling, although they do not yet resolve extracellular-matrix composition at molecular scale. A prospective trial could therefore pair imaging trajectories with a composite pathway panel—such as rigorously defined CD14-positive migrasomes or TSPAN4-positive monocytes together with C5b–9 and established AD/CAA biomarkers—to test whether rising BAM–migrasome activity precedes BBB leakage, declining vascular reactivity, or progressive perivascular injury. Such a design would connect peripheral target engagement to spatially localized neurovascular change and could provide mechanistic end points for early-phase interventions.

Human spatial transcriptomic evidence supports a strong biological distinction between vascular and parenchymal amyloid microenvironments. Chimal-Juárez and colleagues performed spatial whole-transcriptome profiling of postmortem cortical tissue from individuals with mixed AD and cerebral amyloid angiopathy. Regions surrounding vascular Aβ deposits exhibited gene-expression signatures that differed from those surrounding parenchymal plaques. Although both compartments showed extracellular-matrix and matrisome-related responses, the direction and composition of these programs depended on whether amyloid was located within the vessel wall or parenchyma. These observations strengthen the premise that vascular BAM–migrasome signaling represents a spatially specialized mechanism rather than a simple extension of plaque-associated microglial activation. They also indicate that therapeutic effects on parenchymal plaque burden may not predict changes in the vascular extracellular matrix, complement environment, migrasome docking sites, or CAA-related barrier injury [100].

These studies provide the pathological setting in which migrasomes could become important. CAA vessels already show reduced junctional stability, smooth-muscle-cell loss, matrix abnormalities, and local inflammatory activation. A vessel in this state may be especially vulnerable to a macrophage-derived structure that adheres to its surface and concentrates complement-modifying cargo. Conversely, migrasome-induced endothelial damage could be one of the events that converts mild vascular amyloid deposition into progressive leakage, inflammation, remodeling, and hemorrhagic fragility.

### 6.2. Aβ40 Induces Excessive Migrasome Production by Macrophage-Lineage Cells

Hu and colleagues (2023) [57] combined human peripheral samples with CAA mouse models. In the human arm, plasma was analyzed from 12 CAA patients, 12 healthy controls, 28 patients with atherosclerotic cerebral small-vessel disease, 10 with CADASIL, 10 with acute ischemic stroke, and 10 with AD. Plasma migrasomes and the fraction of CD14-positive migrasomes were increased in CAA, while the recruited AD group—selected to exclude MRI/CT evidence of CAA—did not show increased circulating migrasome counts. Monocytes from CAA patients expressed more TSPAN4, TSPAN7, TSPAN9, and NDST1 than controls, and monocyte TSPAN4 correlated with cognitive and MRI measures. Skin-vessel electron microscopy provided human morphological evidence of perivascular migrasome-like structures, but direct human cerebral-vessel or BAM lineage confirmation was not performed [57].

In the mouse brain, TSPAN4- or TSPAN9-positive migrasomes were associated with IBA1-positive macrophage-lineage cells, CD206-positive perivascular macrophages, and infiltrating F4/80-positive macrophages that had internalized Aβ40. Selective depletion experiments indicated that resident perivascular macrophages, microglia, and monocyte-derived macrophages all contributed to the elevated migrasome pool. The findings are therefore relevant to BAM biology but should not be described as demonstrating an exclusively BAM-derived pathway. In CAA, several myeloid lineages encountering vascular Aβ appear capable of producing migrasomes [57].

Aβ40 increased migrasome production in bone-marrow-derived macrophages in a time- and concentration-dependent manner and also stimulated migrasome production in microglia. Inhibition of Aβ uptake reduced this response, showing that surface contact alone was insufficient. Aβ40 internalization increased TSPAN4 expression, and TSPAN4 knockdown reduced migrasome production without preventing Aβ uptake. Conversely, TSPAN4 overexpression increased migrasome generation. This places TSPAN4-dependent membrane organization downstream of Aβ40 phagocytosis and separates amyloid uptake from the subsequent decision to package material into migrasomes [57].

Mechanistic specificity was supported by peptide and inflammatory controls. Aβ40, including oligomerized Aβ40, increased macrophage TSPAN4 expression and migrasome production, whereas Aβ42, Aβ1–16, and Aβ22–35 did not elicit the same response under the tested conditions. Red blood cells did not increase migrasome production, and several inflammatory mediators slightly increased TSPAN4 expression without inducing excess migrasomes. Thus, TSPAN4 up-regulation alone is not equivalent to migrasome production, and the Aβ40 response appears more specific than a generic inflammatory or hemorrhagic response in these experimental systems [57].

What is experimentally known should be separated from the unresolved biochemical link upstream of TSPAN4. The current evidence establishes an order of events: Aβ40 internalization is required for the full response; internalization is followed by increased TSPAN4 expression; TSPAN4 knockdown suppresses excess migrasome production without preventing Aβ40 uptake; and TSPAN4 overexpression increases migrasome output [57]. These perturbations place TSPAN4-dependent membrane organization downstream of Aβ40 uptake, but they do not identify the intracellular sensor, transcriptional regulator, or membrane-remodeling signal that connects an Aβ-containing phagosome/endolysosome to TSPAN4 induction. General migrasome-biogenesis studies implicate TSPAN4–cholesterol membrane domains, sphingomyelin, integrin-dependent adhesion, Rab35, calcium-sensitive membrane expansion, GPI-anchored protein nanoclusters, and retraction-fiber mechanics [43,44,48,49,50,51,52,53]. None of these mechanisms has yet been shown to be the Aβ40-specific upstream link in macrophages. Candidate mechanisms include phagolysosomal stress, redox-sensitive transcription, calcium signaling, lipid/cholesterol remodeling, and changes in actin-dependent migration, but these remain hypotheses. Discriminating among them will require time-resolved promoter/transcriptomic studies, endolysosomal perturbation, redox and calcium manipulation, membrane lipidomics, and live imaging of retraction-fiber/migrasome formation after Aβ40 uptake.

### 6.3. Migrasomes Act as Adhesive Vascular Cargo-Concentration Platforms

Migrasomes are particularly well suited to vascular injury because they combine cargo transport with tissue adhesion. A soluble macrophage protein released into blood or cerebrospinal fluid is rapidly diluted and may never reach a damaging concentration at the endothelial surface. In contrast, an intact migrasome can attach to a vessel, remain at a discrete site, and concentrate its internal or membrane-associated cargo immediately beside endothelial cells.

Aβ40-induced migrasomes readily attached to cultured brain endothelial cells and to brain microvessels after transfer into mice. Disrupting their structure by ultrasound reduced their vascular adhesion and eliminated much of their endothelial cytotoxicity and barrier-disrupting activity, despite preservation of the total concentration of the relevant soluble cargo. The pathological effect therefore depended on an intact organelle capable of docking to the vessel, not simply on the presence of macrophage proteins within the injected preparation [57].

Hu and colleagues (2023) [57] also observed apparent fusion or close membrane interaction between migrasomes and endothelial cells. The exact adhesion molecules remain unknown. Integrins are central to migrasome biogenesis in other systems, but the study did not establish which migrasomal or endothelial proteins mediate attachment in CAA. The vascular extracellular matrix, exposed basement-membrane proteins, endothelial glycocalyx, tetraspanins, phosphatidylserine, integrins, and scavenger receptors are potential candidates requiring direct testing.

This spatial concentration mechanism is important for interpreting circulating CD5L. Plasma CD5L was elevated in the CAA cohort and mouse models, but soluble concentrations were insufficient to reproduce the endothelial injury seen with migrasomes. The authors estimated that migrasome docking increased the local vascular concentration to a biologically damaging range. Thus, systemic biomarker concentration, systemic carrier state, and local tissue exposure are distinct variables. A modest plasma change can coexist with a concentrated molecular microdomain at the vascular wall, and conversely a high circulating concentration does not demonstrate that the same receptor-accessible exposure exists locally [57,85].

### 6.4. CD5L/AIM Links Macrophage Migrasomes to Loss of Complement Resistance

Proteomic analysis identified several complement-associated or inflammatory proteins enriched in Aβ40-induced migrasomes, including CD5L, C-reactive protein, and hemoglobin-related proteins. CD5L—also called AIM—showed the most prominent increase and became the principal mechanistic candidate. Aβ40 increased CD5L expression in macrophages and its abundance within migrasomes. Blocking Aβ internalization or reducing TSPAN4-dependent migrasome formation lowered extracellular-vesicle-associated CD5L, linking amyloid uptake, migrasome biogenesis, and cargo packaging [57].

CD5L-deficient migrasomes lost much of their capacity to activate complement and disrupt endothelial barriers. In macrophage–endothelial co-cultures, Aβ40-stimulated wild-type macrophages caused C5b–9 deposition and ZO-1 loss, whereas deletion of *Cd5l* in the donor macrophages reduced both abnormalities. Reintroduction of CD5L restored injury. Similarly, transferring CD5L-deficient Aβ40-induced migrasomes into mice produced less complement deposition, dextran extravasation, and junctional disruption than transferring CD5L-containing migrasomes [57].

The mechanism did not appear to require CD36, a known CD5L-interacting receptor and an important mediator of Aβ-induced oxidative stress in BAMs. Instead, CD5L reduced endothelial expression of CD59. CD59 is a membrane-bound complement-regulatory protein that prevents assembly of the terminal C5b–9 membrane attack complex. Reduced CD59 lowers the threshold for complement-dependent cytotoxicity, allowing activated complement components to assemble into membrane pores and damage endothelial cells [57].

The migrasomes themselves contained complement proteins, but their levels were not substantially different between control and Aβ40-induced preparations. The damaging effect therefore did not appear to arise simply from delivery of a larger amount of preassembled complement. Instead, the migrasome altered the susceptibility of the recipient vascular surface to the host complement system. CD5L deposition reduced local protection, after which circulating or locally generated complement could assemble into C5b–9.

Human neuropathological findings support the relevance of this terminal complement pathway to CAA. Matsuo and colleagues examined 22 CAA and 12 other neurodegenerative-disease autopsy brains. C1q, C3d, macrophage scavenger receptor, and APOE were strongly associated with capillary Aβ in CAA type 1. C5b–9 and C6 were present in amyloid-affected arteries and arterioles and were preferentially associated with vessels in cases showing subcortical hemorrhage or cortical superficial siderosis. These findings do not identify migrasomes as the source of complement activation, but they demonstrate that terminal complement deposition occurs at clinically relevant CAA vessels [96].

The CD5L–CD59 pathway therefore offers a mechanistic explanation for how complement deposition may become spatially focused. Aβ40-exposed macrophages do not need to synthesize and transport the entire complement cascade. They can instead deposit a cargo that removes a local inhibitory checkpoint, permitting the existing complement system to damage the vessel.

### 6.5. Migrasomes Disrupt Endothelial Survival and Tight-Junction Organization

Aβ40-induced migrasomes produced rapid effects on cultured brain endothelial cells. Transcriptomic analysis showed downregulation of pathways related to cell survival and angiogenesis, together with reduced expression of multiple tight-junction-associated genes, including *Tjp1*, which encodes ZO-1. At the protein level, ZO-1 expression and junctional continuity declined after migrasome exposure [57].

Functional barrier assays confirmed that these molecular changes translated into increased permeability. Endothelial monolayers exposed to Aβ40-induced migrasomes showed reduced transendothelial electrical resistance and increased passage of sodium fluorescein. TSPAN4 knockdown in the macrophage donor protected endothelial ZO-1, while adding isolated Aβ40-induced migrasomes restored the damaging phenotype. This rescue experiment indicated that migrasomes were active mediators rather than incidental markers of macrophage activation [57].

Transfer of Aβ40-induced migrasomes into healthy mice was sufficient to reproduce vascular injury in vivo. The structures accumulated along cerebral vessels, ZO-1 expression declined, and intravenously injected 3 kDa dextran leaked into the brain parenchyma. Plasma neurofilament light increased, suggesting that the vascular insult was accompanied by neuroaxonal injury. White-matter integrity was also impaired, connecting endothelial disruption with tissue-level consequences beyond the immediate vessel wall [57].

These experiments establish sufficiency under the administered conditions, but the transfer paradigm used repeated intravenous doses of isolated macrophage-cell-line-derived migrasomes. It may not reproduce the concentration, route, or kinetics of endogenous migrasomes generated by resident BAMs in human CAA. Moreover, systemic administration does not distinguish luminally circulating migrasomes from structures deposited abluminally by perivascular macrophages. Future lineage-resolved imaging will need to determine which vascular surface is primarily targeted under physiological disease conditions.

### 6.6. CD36–NOX2 Oxidative Stress and TSPAN4–CD5L/AIM Migrasomes Are Parallel, Convergent Pathways

Two BAM-related vascular injury mechanisms should be separated causally. The first is an oxidative-stress branch: Aβ recognition by CD36 activates NOX2-derived ROS in perivascular macrophages/BAMs, producing neurovascular dysfunction; replacing BAM compartments with CD36-deficient cells reduces vascular Aβ40 and CAA and improves vascular reactivity in Tg2576 mice [27,28]. The second is a migrasome–complement branch: Aβ40 internalization increases TSPAN4-dependent migrasome production, CD5L/AIM is enriched within these structures, vascular docking concentrates CD5L/AIM, endothelial CD59 is reduced, and terminal C5b–9-mediated injury is facilitated [57].

The available data do not support a serial CD36 → TSPAN4 → CD5L/AIM pathway. In the 2023 migrasome study, the endothelial complement-enhancing effect of CD5L/AIM was experimentally independent of CD36, whereas TSPAN4 knockdown reduced migrasome production without preventing Aβ40 uptake [57]. Thus, CD36–NOX2 and TSPAN4–CD5L/AIM are best modeled as parallel outputs of amyloid-exposed macrophage-lineage cells that converge on the neurovascular unit rather than as a single verified linear cascade.

Their interaction is nevertheless biologically plausible at the level of convergence and feedback. ROS can disrupt junctions, damage membranes, and lower endothelial reserve, potentially increasing vulnerability to complement-mediated injury; complement activation can recruit myeloid cells and intensify inflammatory/oxidative signaling. Both branches can impair vascular reactivity and barrier integrity, conditions that may reduce perivascular Aβ clearance and increase local Aβ40 retention. This yields a proposed feed-forward model: Aβ40 retention → parallel CD36–NOX2 oxidative stress and TSPAN4–CD5L/AIM migrasome signaling → vascular dysfunction/BBB injury → impaired clearance → further Aβ40 retention.

The cross-talk steps in this feed-forward model remain hypothetical. No study has directly tested whether CD36 or NOX2 manipulation changes TSPAN4 expression, migrasome number, CD5L/AIM sorting, or vascular docking in the same CAA model, nor whether TSPAN4/CD5L manipulation changes BAM ROS production. These experiments are required to distinguish true molecular cross-regulation from downstream convergence of two independent injury programs.

### 6.7. BBB Leakage May Amplify Perivascular Inflammation and Vessel Remodeling

Once endothelial integrity is lost, plasma proteins such as fibrinogen, immunoglobulins, complement components, and coagulation proteins can enter vascular walls, perivascular spaces, and the parenchyma. These substances can activate astrocytes, microglia, BAMs, and recruited monocytes. Barrier disruption may therefore initiate a secondary inflammatory phase even when the first injury was caused by amyloid, oxidative stress, or migrasomes.

The human vessel-grading study by Kozberg and colleagues is consistent with this temporal sequence. BBB leakage and smooth-muscle loss were evident in mildly amyloid-positive vessels, whereas activated microglia and reactive astrocytes became more prominent at later stages. Advanced vessels displayed remodeling features resembling ruptured vessel segments and consistently showed leakage and perivascular inflammation [94].

CAA-associated leakage and microhemorrhage also create a distinct bioinorganic stress environment for perivascular macrophages. Extravasated erythrocytes release hemoglobin, which can generate heme/hemin and redox-active iron after cellular uptake and degradation. Human postmortem CAA microbleeds contain hemosiderin-laden and iron-loaded macrophages and show strong heme oxygenase-1 reactivity around hemorrhagic lesions, demonstrating that macrophages in CAA can experience sustained local heme/iron handling [101]. Macrophage/microglial scavenging through the haptoglobin–hemoglobin–CD163 pathway and heme degradation by HO-1 are protective when detoxification capacity is adequate, with liberated iron normally sequestered by ferritin or exported through ferroportin. When repeated leakage or hemorrhage exceeds this buffering capacity; however, labile heme and Fe^2+^ can amplify Fenton chemistry, membrane and lipid peroxidation, mitochondrial injury, oxidative damage to proteins and DNA, inflammatory signaling, and iron-rich phagolysosomal burden [102]. In long-lived BAMs, such recurrent exposure is therefore a plausible amplifier of metabolic exhaustion, lysosomal dysfunction, and senescence-like reprogramming. This heme/iron route has not yet been tested directly in the TSPAN4–CD5L/AIM migrasome pathway and should be treated as a candidate modifier of CAA-associated BAM dysfunction rather than an established upstream mechanism.

Migrasome-mediated complement activation could contribute to this progression in several ways. Sublytic C5b–9 can alter endothelial transcription and survival without immediately lysing the cell. C5a can recruit and activate myeloid cells. Junctional loss allows additional complement proteins and antibodies to reach the vessel wall. Endothelial death exposes basement-membrane ligands that may further anchor migrasomes and leukocytes. Smooth-muscle-cell loss then weakens the vessel mechanically and impairs vasomotor support. These downstream consequences could transform an initially focal molecular lesion into a larger zone of inflammatory remodeling and hemorrhagic vulnerability.

### 6.8. Relevance to Anti-Aβ Immunotherapy and ARIA

Anti-Aβ antibodies create an additional setting in which vascular amyloid, macrophage activation, complement, and BBB injury converge. Antibody binding to CAA forms immune complexes that activate perivascular macrophages and recruit circulating monocytes. In a 2023 PDAPP mouse study, a murine bapineuzumab analogue bound vascular amyloid and was associated with activation of CD169-positive perivascular macrophages, expression of vascular-permeability and matrix-remodeling genes, plasma-protein extravasation, and monocyte accumulation around microhemorrhages [29].

A subsequent spatial analysis of the same general model showed smooth-muscle-cell loss, BBB disruption, vascular fibrosis, and distinct resident and monocyte-derived TREM2-positive macrophage populations around vascular amyloid after antibody treatment. These findings indicate that therapeutically mobilized Aβ can create a complex perivascular immune response involving several macrophage lineages [77].

A peer-reviewed 2026 study further demonstrated that anti-Aβ antibody binding to CAA rapidly recruited C1q and activated the classical complement pathway. Repeated treatment increased complement deposition, erythrocyte extravasation, microhemorrhages, inflammatory and endothelial-response genes, MMP-9, and BBB disruption. Complement C3 burden correlated with microhemorrhage severity, and perivascular macrophages colocalized with complement-decorated vascular amyloid. These data strengthen the evidence that complement activation can occur upstream of overt ARIA-like vascular injury [103].

Human immunization studies indicate that myeloid responses during antibody-mediated Aβ removal are not uniformly pathological. Van Olst and colleagues used spatial transcriptomics to compare actively immunized patients with AD, non-immunized patients with AD, and neurologically healthy controls, and supplemented this analysis with high-resolution profiling of tissue from a lecanemab-treated patient. Immunization was associated with spatially and regionally distinct microglial states linked to Aβ clearance. APOE- and TREM2-associated programs were increased across active and passive immunization approaches and correlated with antibody responses and local Aβ removal. These findings argue against interpreting every treatment-associated myeloid response as evidence of toxicity. Instead, the outcome may depend on the anatomical compartment and functional state of the responding cells: plaque-associated microglial activation may support Aβ removal, whereas excessive activation of complement at amyloid-laden vessels may promote barrier injury and hemorrhagic complications [104].

Accordingly, the proposed CD5L/AIM–complement pathway should be framed as a disturbance of spatial and regulatory control rather than as evidence that complement and myeloid activation are globally detrimental. A successful intervention may need to suppress vascularly concentrated terminal-complement injury while preserving microglial immune-complex handling, TREM2-dependent responses, and antibody-mediated Aβ clearance.

No study has yet demonstrated that migrasomes cause amyloid-related imaging abnormalities. The connection is therefore mechanistically plausible but unproven. Antibody treatment could increase macrophage migration, Aβ uptake, Fc-receptor signaling, and complement exposure, all of which might affect migrasome number or cargo. Conversely, CD5L-rich migrasomes could reduce vascular complement resistance precisely when antibody–CAA complexes are activating the classical pathway. This potential interaction should be tested directly by measuring TSPAN4-positive migrasomes during anti-Aβ treatment and determining whether TSPAN4 or CD5L manipulation changes ARIA-E or ARIA-H.

### 6.9. Human Biomarker Evidence: Promising Signal, Insufficient Validation

The strongest human biomarker signal currently comes from the same small CAA cohort that generated the mechanistic hypothesis. The percentage of TSPAN4-positive peripheral monocytes discriminated 12 CAA patients from 12 healthy controls with an exploratory ROC AUC of 0.9097 ± 0.0590 (*p* = 0.0007), and monocyte TSPAN4 was associated with cognitive and MRI indicators of CAA [57]. This result is encouraging but does not establish clinical sensitivity or specificity: no externally validated decision threshold, confidence intervals for threshold-specific sensitivity/specificity, prospective replication cohort, or assay-harmonization study has been reported. TSPAN4 is also a membrane tetraspanin expressed outside migrasomes, so TSPAN4 positivity should be interpreted as a component of a migrasome-producing myeloid phenotype rather than as a stand-alone proof of migrasomes.

The same study found increased total circulating migrasomes and a selectively increased CD14-positive migrasome fraction in CAA compared with healthy controls and the recruited neurological comparator groups, including AD without imaging evidence of CAA [57]. This pattern suggests potential CAA enrichment, but the sample sizes were small and the isolation/flow-cytometric definition of migrasomes has not been independently standardized. A clinically deployable assay will need blinded external validation, predefined particle-identification criteria, reproducibility across collection/processing protocols, and comparison against updated CAA diagnostic standards and MRI markers.

CD5L/AIM is biologically informative but currently weaker as a stand-alone disease marker. Plasma CD5L/AIM was increased in the CAA cohort, yet the original investigators explicitly concluded that it lacked diagnostic value because CD5L/AIM rises in multiple injurious conditions [57]. Human CSF work indicates that CD5L/AIM is present predominantly in an IgM-associated form, changes with aging, and did not show a significant AD-specific difference in the small AD versus no-cognitive-impairment comparison reported by Shuken et al. [105]. An amyloid-PET study found reduced CD5L/AIM in an albumin-associated plasma fraction but unchanged whole-plasma CD5L/AIM [106], while a two-cohort plasma-proteomics study found inconsistent directions of CD5L change across clinical cohorts [107]. Collectively, these data argue strongly against using soluble CD5L/AIM alone as an AD- or CAA-specific biomarker.

A more defensible translational strategy is therefore a composite, compartment-aware biomarker model rather than a single-analyte claim. Candidate panels should combine a macrophage-lineage signal (for example TSPAN4-positive monocytes or rigorously defined CD14-positive migrasomes), a pathway-effector readout such as C5b–9, and established disease context such as MRI-defined CAA burden and core AD biomarkers. Sensitivity, specificity, positive/negative predictive values, calibration, and incremental value over MRI and established plasma/CSF markers should be estimated in independent, adequately powered cohorts. Until such validation is available, TSPAN4 and CD5L/AIM should be described as mechanistically motivated candidate biomarkers, not clinically validated diagnostic markers.

Translation of circulating migrasomes into a clinical biomarker will additionally require rigorous pre-analytical and analytical standardization. Sample collection, anticoagulant choice, processing delay, centrifugation, freeze–thaw cycles, and storage conditions may alter recovery of large extracellular particles. Because migrasomes overlap in size and marker expression with other extracellular vesicles and apoptotic bodies, TSPAN4 positivity or particle size alone is insufficient for identification. Future studies should therefore combine orthogonal approaches, including high-resolution imaging or imaging flow cytometry, particle-size analysis, and multiparametric marker panels together with morphological criteria, in accordance with contemporary extracellular-vesicle reporting principles such as MISEV2023. Translational status of candidate markers linked to the BAM–migrasome pathway is presented in Table 2.

### 6.10. Targeted Therapeutic Strategies and Clinical Operability

The clearest proof of principle remains downstream complement inhibition. In endothelial cultures, PMX-53, avacopan, and eculizumab reduced injury caused by Aβ40-induced migrasomes; in mice, PMX-53 reduced C5b–9, preserved ZO-1, limited BBB leakage, and reduced white-matter injury [57]. The peer-reviewed anti-Aβ immunotherapy study showing early complement activation at CAA provides an additional rationale for testing whether carefully timed complement modulation can reduce vascular toxicity during amyloid mobilization [103]. However, systemic complement blockade can impair host defense and immune-complex handling, so chronic use in AD/CAA requires formal safety and efficacy testing.

Upstream targeting may provide greater pathway selectivity but should avoid global inhibition of BAM function. Systemic TSPAN4 blockade is not yet clinically justified because TSPAN4 is not unique to pathological migrasomes and migrasome biogenesis occurs in multiple tissues. A more operable preclinical strategy would use lineage- or niche-targeted delivery—such as nanoparticles or other vectors preferentially taken up by perivascular/macrophage populations—to reduce excessive TSPAN4-dependent migrasome production only in amyloid-exposed cells. Target engagement should be verified by reduction in rigorously defined circulating or tissue migrasomes rather than by TSPAN4 expression alone.

Precision staging may be equally important as anatomical targeting. Because BAMs appear to move from clearance-competent to metabolically exhausted or senescent-like states along a continuum, trial eligibility and treatment timing could be guided by longitudinal multimodal profiles rather than diagnosis alone. A practical framework would integrate CAA/BBB and neurovascular imaging, APOE genotype and vascular risk, composite plasma/CSF pathway markers (for example, migrasome/TSPAN4 signals plus C5b–9), and repeated digital phenotyping. Wearable or portable digital biomarkers in early AD most often capture activity/rest, speech, gait, and related functional domains, but their prognostic and treatment-monitoring validity remains limited [108]; they should therefore be used as complementary temporal readouts rather than BAM-specific markers. In principle, within-person changes in these digital measures could help flag clinically silent transitions for confirmatory imaging and molecular sampling, enabling local TSPAN4-, migrasome-docking-, or complement-directed therapy to be deployed after loss of clearance reserve but before fixed hemorrhagic or senescence-associated injury. This stage-enriched approach is more consistent with preserving beneficial BAM clearance than indiscriminate depletion or lifelong pathway blockade.

CD5L/AIM requires particular caution as a target. In the CAA model, migrasome-localized CD5L/AIM was necessary for complement-dependent BBB injury [57], but a 2026 peer-reviewed AD study showed that CD5L/AIM can bind Aβ, reduce aggregation, enhance microglial Aβ uptake, reduce plaque burden, and improve cognition in 5xFAD mice [84]. These apparently opposite effects are compatible if CD5L/AIM function depends on compartment, molecular form, concentration, and presentation. Consequently, global CD5L/AIM neutralization could remove beneficial Aβ-handling effects. More selective options include preventing pathological loading or vascular docking of CD5L/AIM-rich migrasomes, preserving/restoring endothelial CD59, or locally limiting terminal complement activation at CAA-positive vessels.

For clinical translation, future intervention studies should enroll well-phenotyped CAA or AD-with-CAA populations, stratify APOE genotype and vascular risk, and pair safety MRI endpoints (ARIA-E/ARIA-H, microbleeds, siderosis, BBB permeability) with pathway pharmacodynamic markers (TSPAN4-positive monocytes, validated migrasome counts, C5b–9, and established AD biomarkers). The first objective should be target engagement and vascular safety rather than broad cognitive efficacy. Only after human biomarker reproducibility and target engagement are established should TSPAN4-, migrasome-docking-, CD59-, or complement-directed strategies be advanced as disease-modifying approaches.

## 7. Conclusions

The most defensible current model is a BAM-centered vascular and neuroimmune feed-forward axis rather than a fully established linear pathway in human AD. Peer-reviewed experimental evidence supports two parallel consequences of amyloid exposure: a CD36–NOX2 oxidative-stress branch that impairs neurovascular function and an Aβ40–TSPAN4–CD5L/AIM migrasome branch that lowers endothelial complement resistance and promotes BBB injury. Aging experiments further show that CD5L/AIM-rich BAM migrasomes can transmit apoptosis resistance and senescence-like dysfunction to microglia [27,28,57,64]. Direct molecular cross-regulation between the oxidative and migrasome branches has not been demonstrated; their proposed interaction currently rests on convergence at the neurovascular unit and on a plausible feedback through impaired clearance and additional Aβ40 retention. The vascular branch is most strongly supported for CAA and AD with prominent vascular Aβ40, whereas its operation in Aβ42-dominant parenchymal AD without substantial CAA remains unverified [68,69,70].

Human translation remains the principal limitation. The strongest clinical evidence comes from a small CAA cohort with increased circulating macrophage-lineage migrasomes and monocyte TSPAN4, plus peripheral skin evidence of CD5L/AIM-containing migrasome-like vascular deposits [57]. Direct demonstration of BAM-derived migrasomes in human AD brain tissue is absent, and AD patients selected to lack imaging evidence of CAA did not show increased circulating migrasome counts in that study. TSPAN4 therefore remains a candidate component of a CAA-focused biomarker panel rather than a validated migrasome-specific marker, while soluble CD5L/AIM lacks adequate disease specificity and may have context-dependent beneficial as well as harmful actions [57,84,105,106,107]. Similarly, the microglial recipient mechanism should be described as CD5L/AIM-dependent and CD16-associated: direct CD5L–CD16 binding has not been established, and the available Fcγ-receptor blockade is not CD16-exclusive [64,86,87].

The next decisive studies should independently replicate the human CAA findings, compare AD with prominent CAA against biomarker-defined AD without CAA, identify migrasomes directly in human cerebral vascular/border compartments with multiparametric criteria, establish the producing BAM lineage, and measure both CD36–NOX2 activity and TSPAN4–CD5L/AIM signaling in the same disease models. Aβ40 and Aβ42 should be compared directly as upstream stimuli, and the intracellular pathway linking Aβ40 uptake to TSPAN4 induction should be resolved. Receptor-level studies should test Fcgr3/CD16 genetically, distinguish FcγRIII from FcγRII, and determine whether CD5L physically binds the receptor. Biomarker studies should report prespecified thresholds, sensitivity, specificity, calibration, and incremental value over MRI and established AD markers. Therapeutic development should prioritize local or pathway-selective interventions that preserve clearance-competent BAMs while suppressing vascular complement amplification or pathological migrasome deposition. Until these steps are completed, the BAM–migrasome axis is best regarded as a mechanistically coherent and testable framework with the strongest current relevance to CAA and AD-with-CAA, but not a validated universal mechanism or biomarker system for sporadic AD.

## Figures and Tables

**Table 1:** Experimental and translational evidence supporting the proposed BAM–migrasome axis.

Principal Limitation	Evidence Level	Main Finding Relevant to Proposed Axis	Model/Material	Study/Evidence Source
No demonstration of BAM-derived migrasomes in human brain; small human cohort	Experimental causal evidence + human-associated observations	Aβ40 internalization → TSPAN4-dependent migrasomes enriched in CD5L/AIM → reduced endothelial CD59 → complement-mediated vascular injury	macrophage/microglial cultures, mouse CAA models, small human CAA cohort	Hu et al. (2023) [57]
Aging rather than AD model; same research group as [57]; no independent replication	Experimental causal evidence	senescent-like BAM migrasomes transfer CD5L/AIM and induce CD16-associated apoptosis resistance/senescence-like dysfunction in microglia	physiological aging in mice + cell experiments	Hu et al. (2025) [64]
Supports parallel oxidative branch, not direct migrasome mechanism	Experimental causal evidence	Aβ–CD36–NOX2 signaling contributes to vascular oxidative stress	AD/CAA mouse model	Uekawa et al. [28]
Does not demonstrate migrasome production or causal sequence	Human observational evidence	Human BAM populations and AD-associated BAM alterations are present	postmortem/scRNA/spatial analyses	Human AD/BAM profiling studies
Preprint; not independently validated	Provisional	progressive loss/metabolic impairment of BAM2-like state	5 × FAD + human datasets/tissue	BAM2 preprint [40]

**Table 2:** Translational status of candidate markers linked to the BAM–migrasome pathway.

Candidate Readout	Human Evidence	Current Interpretation/Required Validation
TSPAN4-positive monocytes	CAA *n* = 12 vs. healthy controls *n* = 12; exploratory AUC 0.9097 ± 0.0590; correlated with cognitive/MRI severity [57].	Promising CAA signal, but no externally validated cutoff or threshold-specific sensitivity/specificity. TSPAN4 is not migrasome-specific.
CD14-positive circulating migrasomes	Increased in CAA and not increased in recruited AD without imaging CAA, AIS, CADASIL, or atherosclerotic CSVD groups [57].	Potential CAA enrichment; requires independent replication and standardized particle isolation, morphology, marker panel, and flow-cytometric gating.
Plasma/CSF CD5L/AIM	Plasma increased in small CAA cohort [57]; CSF CD5L-IgM varies with aging [105]; recent human AD plasma studies show compartment- and cohort-dependent results [106,107].	Insufficient specificity as a soluble stand-alone marker; interpret together with migrasome localization and disease context.
C5b–9	Plasma increased in CAA and showed favorable exploratory ROC discrimination from controls and selected small-vessel-disease comparators [57].	Mechanistically downstream and not migrasome-specific. Useful as a pathway-activity component, not proof of BAM-derived migrasomes.

## Data Availability

No new data were created or analyzed in this study. Data sharing is not applicable to this article.
